# *In vitro* synthesis of gene-length single-stranded DNA

**DOI:** 10.1038/s41598-018-24677-5

**Published:** 2018-04-25

**Authors:** Rémi Veneziano, Tyson R. Shepherd, Sakul Ratanalert, Leila Bellou, Chaoqun Tao, Mark Bathe

**Affiliations:** 1 Department of Biological Engineering, Massachusetts Institute of Technology, Cambridge, MA 02139 USA; 2 Department of Chemical Engineering, Massachusetts Institute of Technology, Cambridge, MA 02139 USA

## Abstract

Single-stranded DNA (ssDNA) increases the likelihood of homology directed repair with reduced cellular toxicity. However, ssDNA synthesis strategies are limited by the maximum length attainable, ranging from a few hundred nucleotides for chemical synthesis to a few thousand nucleotides for enzymatic synthesis, as well as limited control over nucleotide composition. Here, we apply purely enzymatic synthesis to generate ssDNA greater than 15 kilobases (kb) using asymmetric PCR, and illustrate the incorporation of diverse modified nucleotides for therapeutic and theranostic applications.

## Introduction

Efficient ssDNA synthesis on the 10+ kb-scale is a major need for numerous biotechnology applications including templated homology directed repair for genome editing^1–4^, systems-scale gene synthesis and cloning^5–9^, and scaffolded DNA origami^10,11^. Conventional ssDNA synthesis is performed using either chemical or enzymatic approaches. Chemical synthesis is currently limited to approximately 98% incorporation efficiency for each base addition and is therefore limited to the production of ssDNA oligonucleotides up to only 200 bases^5^. Subsequent enzymatic synthesis through ligation or polymerization yields double-stranded DNA (dsDNA), enabling complete gene synthesis that can be further parallelized^12^, but requires additional steps to generate ssDNA. Alternatively, direct synthesis of longer ssDNA from oligonucleotides can be accomplished by primer exchange reaction^13^ or can be obtained from commercial sources up to 2 kb from Integrated DNA Technologies, Inc. (IDT, Coralville, IA) or recommended up to 5 kb using a strandase enzyme-based approach from Takara Biosciences, Inc. (Mountain View, CA). Enzymatic or chemical approaches to denature dsDNA to form ssDNA are alternative approaches to ssDNA production, but are often limiting in purification strategies^14–16^. Large-scale bacterial production of pure ssDNA of near arbitrary sequence is achievable using engineered plasmids^17^ or helper M13-*Escherichia coli* strains in combination with phagemids^18,19^, but is complicated by the necessity of ssDNA origin-of-replication and selection-gene sequences and usage of canonical deoxynucleotides.

In contrast, asymmetric polymerase chain reaction (aPCR) offers the direct synthesis of ssDNA from an underlying dsDNA template, unlinked to biological replication sequences, and has been applied to generate ssDNA ranging from several hundred to several thousand nucleotides in length^20–23^. aPCR differs from traditional PCR by having one primer (the forward primer) in molar excess over the second primer (the reverse primer). This approach has previously been applied to short ssDNA synthesis for aptamers and gene detection^20,23^, and more recently to kb-scale ssDNA for scaffolded DNA origami^11^. However, previous work was limited to 3.3 kb due to low enzyme processivity. Here, we overcome this limitation by using a highly-processive LongAmp Taq polymerase to achieve 15+ kb length ssDNA. Additionally, using a standardized protocol and rules-based primer design, we achieve pure product yields up to 690 ng per 50 µL reaction volume (2 pmoles for a 1,000 nt ssDNA fragment) and demonstrate direct incorporation of chemically modified nucleotides for ssDNA applications in therapeutics and theranostics that require base or backbone modifications.

## Results

**Figure 1:**
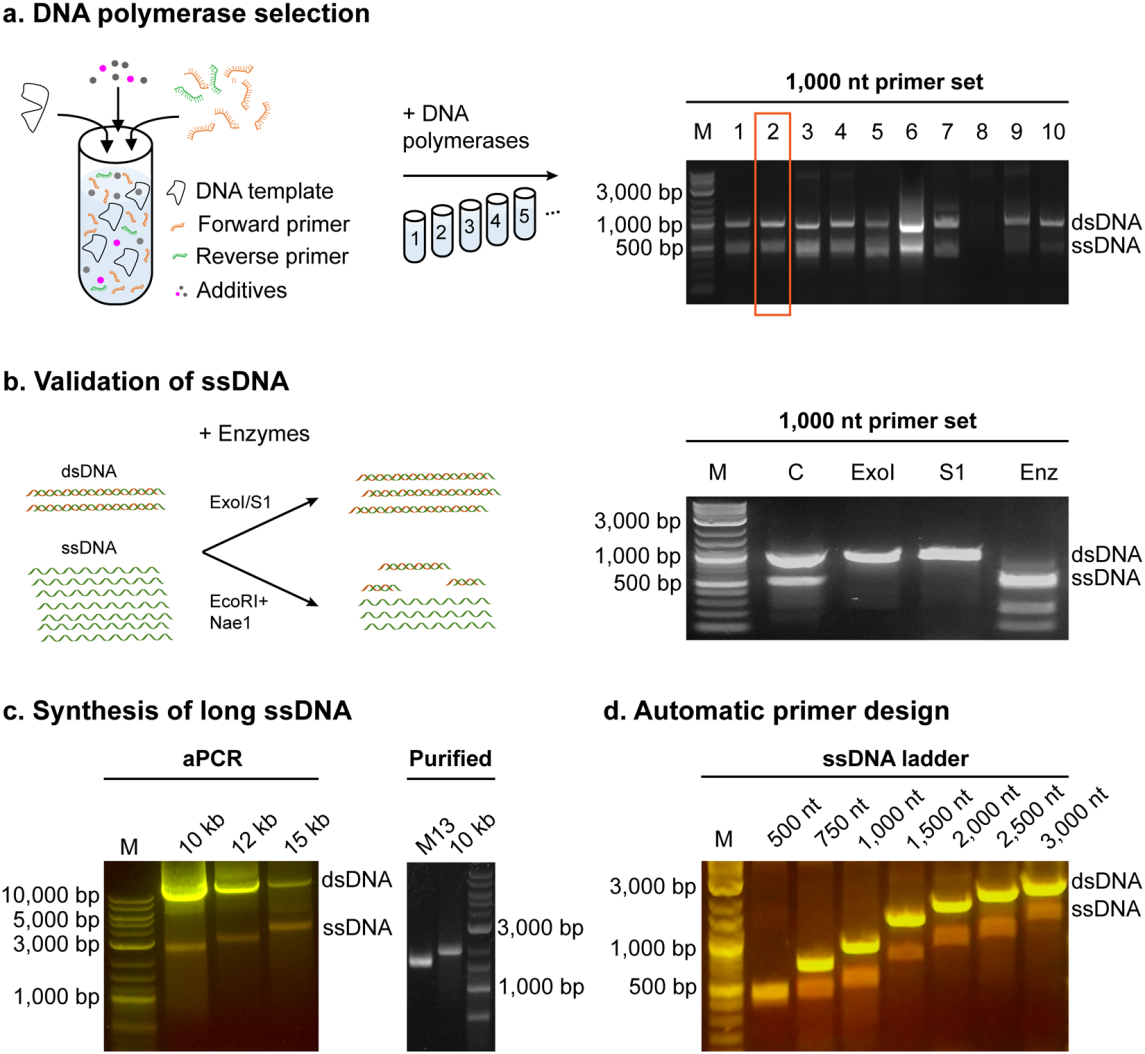
ssDNA production by aPCR. (**a**) aPCR reactions were assembled with a 50-molar excess of a forward primer for the amplification of a 1,000 nt ssDNA fragment using the M13mp18 ssDNA plasmid as template, and with 10 different polymerases that were tested for highest yield of ssDNA production (upper band: expected dsDNA size is 1,000 bp; lower band: expected ssDNA size is 1,000 nt) as judged by agarose gel electrophoresis (right panel). QuantaBio AccuStart HiFi, polymerase (lane 2, boxed) produced the highest amount without overlapping dsDNA contaminants. 1. Accustart; 2. Accustart HiFi; 3. Accustart II; 4. AccuPrime; 5. GoTaq; 6. DreamTaq; 7. Phusion; 8. Platinum SuperFi; 9. Q5; 10. Tth polymerase. (**b**) Biochemical validation of ssDNA production by incubating 1,000 nt aPCR reaction products with the ssDNA-specific ExoI or S1 nucleases or dsDNA-specific restriction enzymes *Eco*RI and *Nae*I (left panel). Agarose gel electrophoresis of the digestion products as labeled by lane (right panel). M: Marker, C: aPCR product control, ExoI: exonuclease I, S1: S1 nuclease, Enz: *Eco*RI + *Nae*I. (**c**) NEB LongAmp was used to generate ssDNA up to 15,000 nt long using lambda phage dsDNA as template. Purification of the 10 kb fragment shows a single band of higher molecular weight than the M13mp18 ssDNA (7,249 nt). (**d**) The primer design algorithm aPrime was used to select primers for product sizes between 500 and 3,000 nt using M13mp18 ssDNA as template and the Quantabio Accustart HiFi enzyme. SYBR Safe stained agarose gels illuminated under blue light show dsDNA as yellow bands, while ssDNA show as orange bands.

High-fidelity polymerases such as Phusion® allow for long dsDNA synthesis in standard PCR; however, Phusion polymerase was unable to synthesize fragment larger than 1 kb ssDNA (Fig. 1a, lane 7 and S1, lane 7), likely due to enhanced exonuclease activity as observed with commercial genetically engineered Deep Vent (exo-) polymerase without 3′ → 5′ proofreading exonuclease activity (Fig. S2). Thus, we evaluated 10 polymerases, representative of different families of enzymes, for their activity in generating ssDNA (Figs 1a and S1, Supplementary Table S1). Based on agarose gel band intensities, *Taq* polymerases gave the highest ssDNA production (Fig. 1a, Lane 1–6; S1, Lane 1–6; all quantified gel intensities can be found in External Table S1), possibly due to lower exonuclease activity. QuantaBio AccuStart HiFi yielded the largest amount of ssDNA with the fewest contaminating dsDNA off-target strands for both 1,000 nt (Fig. 1a, Lane 2 boxed and External Table S1) and 3,281 nt fragments (Fig. S1, Lane 2 and External Table S1). To ensure single-strandedness of the product, the reaction was incubated with S1 and *Exo*I nucleases, both specific to ssDNA, showing only digestion of the low molecular weight band (Fig. 1b), while using dsDNA-specific restriction endonucleases (*Eco*RI + *Nae*I) showed digestion of the dsDNA high molecular weight band (Fig. 1b, lane Enz). *Taq* polymerase was capable of synthesizing ssDNA from natural templates such as M13mp18 ssDNA plasmid, dsDNA amplified product (Fig. S3a) and from templates synthetic in origin such as from a digital information-encoding sequence (Fig. S3b,c and Supplementary Table S2). A general protocol was developed for the highest product yield, specific to AccuStart HiFi polymerase, showing 2 mM MgSO_4_ concentration, 1:50 to 1:65 reverse:forward primer ratio, 0.6 ng/µL template concentration, and up to 40 cycles (Figs S4 and S5; External Table S1), with gel extraction being used for subsequent purification (Fig. S6) yielding an average synthesis in the 1 kb range of 695 ± 35 ng (2 pmoles for a 1,000 nt ssDNA fragment) of long ssDNA per 50 µL of aPCR reaction (Fig. S7 and External Table S2). We validated the reproducibility of our approach by running multiple replicates of different ssDNA lengths (Fig. S8 and External Table S2).

AccuStart HiFi was capable of synthesizing ssDNA up to 6,000 nt, but with reduced yield (Figs S9 and S10, Supplementary Table S3, and External Table S2). Initial tests with two other *Taq*-based polymerase sets, NEB LongAmp® *Taq* and Takara LA® *Taq*, produced notable amounts of dsDNA byproduct when tested for amplification of the 1,000 nt and the 3,281 nt fragments and reduced amount of ssDNA per reaction for the 1,000 and 3,281 fragments respectively in comparison with the Accustart HiFi (Fig. S11 and Supplementary Table S4, External Table S1). However, these byproducts were avoided by increasing the annealing temperature (Fig. S11). Given the capacity for these polymerases to synthesize long dsDNA fragments, these enzymes were tested for use in 10+ kb-length ssDNA synthesis. Lambda phage genomic DNA (New England BioLabs Inc., NEB) was used as a template for long-strand synthesis, with the protocol being only slightly modified, including using less template (0.01 to 0.5 ng/µL) and increasing the extension time commensurate with the product length. With these modifications, the LongAmp and the LA *Taq* enzymes were capable of producing ssDNA products 10, 12, and 15 kb in length (Figs 1c and S10, S12–S14). While both of the enzymes were capable of synthesis of long fragments, the NEB LongAmp gave the highest yield according to gel band intensity and was reproducibly purified (50 fmoles, 20 fmoles, and 90 fmoles per 50 µL of reaction for the 10, 12, and the 15 kb, respectively) (Figs S12–S14, External Table S2).

To ensure highest yield of user-defined product lengths, primer design rules were generated to reduce off-target sequence amplification, as exponential amplification of undesirable off-target dsDNA will exceed target linear ssDNA production. These include the forward and reverse primers not priming at off-target sequences on the template or product. Additionally, similar to LATE-PCR, the melting temperature of the forward primer should be 1–3 °C less than the melting temperature of the reverse primer due to the higher concentration of the former. To reduce mispriming, the forward primer should be more GC-rich in the 5′ half than the 3′ half, and the 3′-nucleotide should terminate in an A or T. Additionally, we found highest ssDNA yield when the forward primer melting temperature is between 54 and 57 °C. We codified the aPCR-specific rules into an algorithm for rapid retrieval of application-specific primer sets for user-selected product lengths (named “aPrime”) and experimentally validated the algorithm for products ranging from 500–15,000 nt (Figs 1c,d, S15 and Supplementary Table S5). Notably, additional template constraints such as limiting high GC-content and avoiding long regions of sequence self-similarly should be avoided.

**Figure 2:**
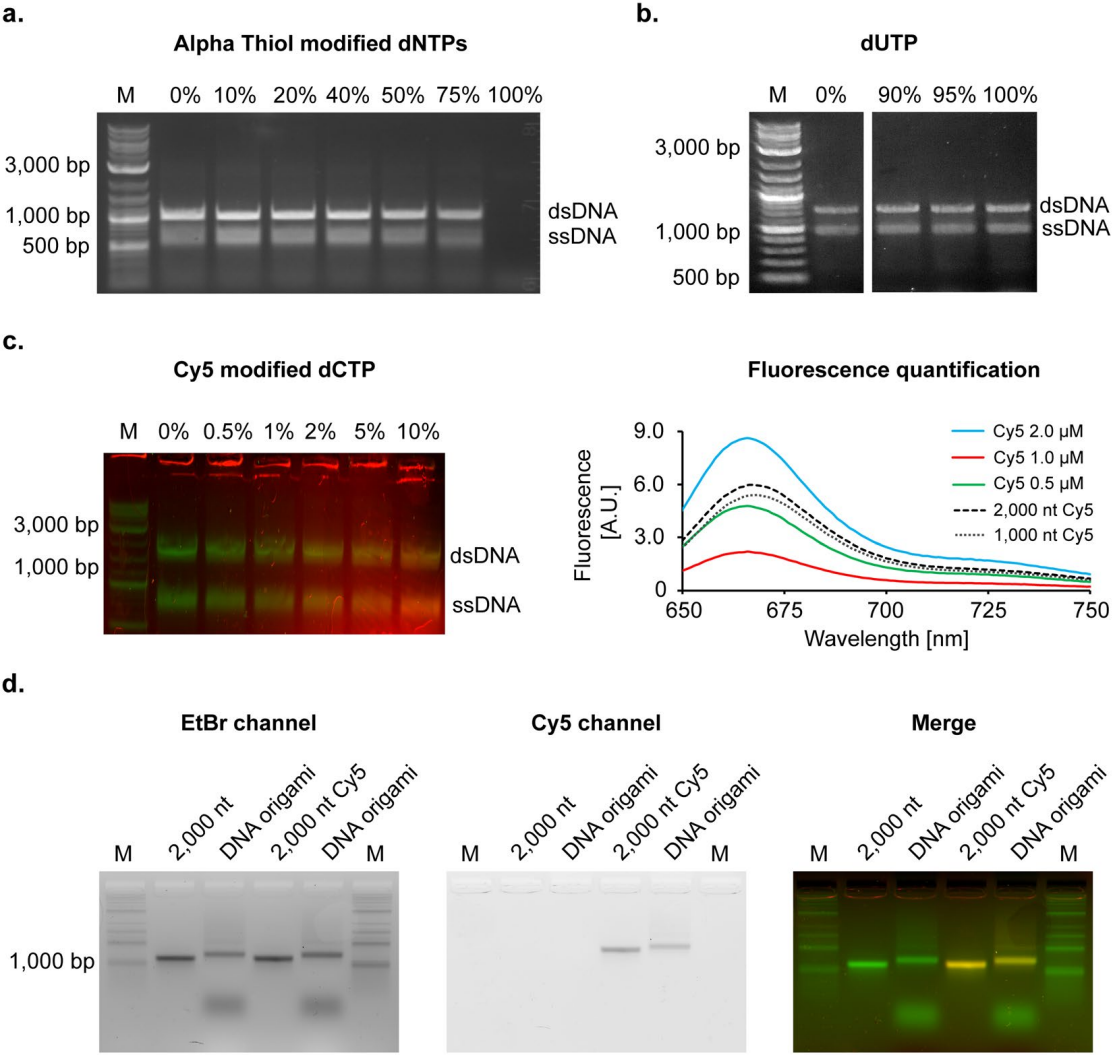
Chemically modified ssDNA produced by aPCR. Accustart Taq polymerase HiFi is able to incorporate various chemically modified dNTPs into ssDNA produced with aPCR. (**a**) Triphosphate-modified nucleotide incorporation is realized by replacing canonical dNTPs with varying concentration of modified alpha-thiol dNTPs (4 bases). ssDNA production is effective for a replacement of dNTPs up to 75% (upper band: dsDNA 1,000 bp; lower band: ssDNA 1,000 nt). See Fig. S16 for higher percentage of dNTPs replacement. (**b**) dUTP replacement for dTTP up to 100% in asymmetric production of the ssDNA. Different parts of the same gel and exposure are shown and the split indicated by a white space. See Fig. S17 for the uncropped gel. (**c**) Cy5 fluorescently modified dCTP can be used to up to 10% replacement of canonical dCTP to be incorporated into the synthesized ssDNA (left). Merged image of ethidium bromide and Cy5 channels. See Fig. S19 for the full ethidium bromide- and Cy5-channel images. The efficiency of incorporation of Cy5-dCTP is quantified by fluorimetry and show approximately 2.5% modification of the Cy5 in ssDNA when 5% of Cy5-modified dCTP are used in aPCR for both 1,000 and 2,000 nt fragments (right). (**d**) Fluorescently modified ssDNA 2,000 nt is used to fold DNA nanoparticles. DNA stain and fluorescent agarose gel mobility shift assay showing folding of a fluorescent scaffolded DNA origami nanoparticle compared to non-fluorescent DNA nanoparticles.

We extended the capabilities of single-strand synthesis to incorporate modified dNTPs dispersed throughout the entire polymer for therapeutic and theranostic or imaging applications, similar to what has been shown in dsDNA synthesis^24,25^. We tested this strategy using four different dNTPs, replacing in varying percentages one or all four of the canonical dNTPs. As phosphorothioates are used for nucleic acid polymer stability in the presence of nucleases, we evaluated the efficiency of their incorporation into ssDNA by titrating bulk dNTP phosphorothioate concentration ratios from 0 to 100%. Yield decreased with higher percentage of modified dNTPs to a limit of ~75% before synthesis failed or stalled (Figs 2a and S16, External Table S2). To test base modification incorporation, we next tested dUTP incorporation into single-stranded DNA synthesis as a replacement for thymidine triphosphate (dTTP). Using pre-generated and purified template generated with 100% dTTP to limit template mutations, we synthesized a gene-length product using complete replacement of dTTP with dUTP (Figs 2b and S17). For application in molecular coordination, we additionally incorporated biotinylated dNTPs into the synthesized strand (Fig. S18, External Table S2). For applications in fluorescence imaging, we evaluated the synthesis of ssDNA with direct incorporation of Cy5-modified dCTP, with up to 10% modified nucleotides (Figs 2c and S19). Subsequent gel purification and quantification using fluorimetry showed up to 2.5% total incorporation on two different templates (1,000 and 2,000 nt) while using 5% of modified Cy5-dCTP in the dNTP mix (Fig. 2c, right panel) with a similar yield of ssDNA than the non-modified dNTPs (External Table S2). Purified fluorescent ssDNA was used as a scaffold to fold open wireframe DNA origami nanoparticles (a pentagonal bipyramid with a 2,000 nt scaffold and a tetrahedron using a completely synthetic data-encoding 1,087 nt sequence; Figs 2d, S20, and External Table S3), showing that the chemical modification does not disrupt folding, and can be used for further fluorescent tracking of particles in downstream *in vitro* and *in vivo* biodistribution assays. Additional modification of 10 kb length ssDNA with Cy5-dCTP was also demonstrated, showing long-strand synthesis of fluorescent polymer can be achieved (Fig. S21). Thus, this work demonstrates the capability of aPCR to produce a variety of useful chemically modified ssDNAs, in addition to 100% custom sequence.

In this work, we have extended a simple method for generating ssDNA using aPCR, which allows for robust synthesis up to 15 kb and additionally allows for one pot chemical modification of ssDNA. The capabilities introduced here will enable future biotechnological applications, including insertion of large chemically- or fluorescently-modified gene constructs through single gene editing experiments, long ssDNA-based digital memory storage, and scaffold-modified structured nanoparticle synthesis, amongst others.

## Methods

### Computational strategy for primer design

A primer design algorithm was implemented to codify rules for single-stranded DNA production using aPCR. Primer annealing temperature was calculated using the nearest-neighbor model with sodium and magnesium salt corrections^26–28^. The template sequence from either lambda or M13 phage genomic DNA (NCBI: NC_001416 and M77815, respectively) or arbitrary, user-based sequences are specified as input together with the size of the desired final amplified product. Sets of primers are first identified satisfying the following rules for forward primer design: (1) primer length between 18 and 22 nt, inclusive; (2) melting temperature between 54 °C and 57 °C; (3) guanine-cytosine (GC) content between 40 and 60%; (4) local GC-content near 3′ end less than that of the 5′ end; and (5) terminating the 3′ end in an A or a T. Reverse primer sequences are generated by shifting the window downstream; the length of the final amplified product is bounded by the outermost ends of the primers. Potential reverse primer sequences leading to this position are tested against a second distinct set of rules: 6) primer length between 18 and 22 nt; (7) melting temperature between 1 and 3 °C higher than that of the forward primer, similar to LATE-PCR^29^; (8) GC-content between 40 and 60%; and (9) terminating the 3′ end in a C or a G. Primer pairs that satisfy these rules are tested for alternative possible priming sites by Blast 2.6.0+ ^30^ and are excluded if the primer has homology to any other location on the template greater than 9 nt (word size = 9). Selected primers passing all of the aforementioned tests are provided as output as potential pairs that would yield the product of the defined size. All primer pairs satisfying these rules for M13 and lambda phage genomes of all possible sizes per template are stored in a database for rapid access. Additionally, all experimentally validated primers are noted in the database. The software is implemented as open source in Python 3.4 and SQLite3 under GPL v2.0, with an associated web-based portal available at http://daedalus-dna-origami.org/aPrime/.

### Polymerase enzyme characterization for ssDNA production

To evaluate reaction conditions for the synthesis of long ssDNA, two sets of validated aPCR primers and M13 genomic ssDNA as a template were used to generate products of sizes 1,000 nt and 3,281 nt (Table 1)^11^.

**Table 1:** Primers used to investigate aPCR synthesis conditions.

**Fragment sizes (nt)**	**Forward primer sequence**	**Reverse primer sequence**
1,000	GTCTCGCTGGTGAAAAGAAA	ATTAATGCCGGAGAGGGTAG
3,281	TCTTTGCCTTGCCTGTATGA	GCTAACGAGCGTCTTTCCAG

The following enzymes were purchased from the respective commercial providers to test enzymatic production of ssDNA: AccuStart™, AccuStart™ II, and AccuStart™ HiFi from Quantabio; Q5® hot start HiFi, Phusion®, LongAmp®, Deep Vent®, and Deep Vent® (exo-) from New England BioLabs Inc. (NEB); AccuPrime™, Platinum™ SuperFi™, Tth and DreamTaq™ from ThermoFisher Scientific Inc.; GoTaq® from Promega (Promega corp.); and LA *Taq*® from Takara Bio. These were characterized for their aPCR efficiency, yield, and off-target production metrics for the quality of aPCR. Each enzyme was evaluated using the supplier’s recommended protocol for traditional PCR, including supplied buffers (Supplementary Table S1), with the exception that the forward primer was used in 50-fold excess over the reverse primer. Annealing temperatures were evaluated at 55 °C and reactions were thermocycled 30 times. Products were run on a 0.7–1% low-melt agarose gel (IBI Scientific, Inc.) cast with ethidium bromide (Sigma Aldrich, Inc.) or SybrSafe (ThermoFisher Scientific, Inc.) at 90 V for 1–2 hours, depending on fragment size, and visualized under blue or UV light illumination. Equal reaction volumes were used in each lane. Band intensities were analyzed with ImageJ Gel Analyzer^31,32^ for comparison.

### Reagent evaluation

Systematic evaluation of the aPCR reaction included characterizing the impact of magnesium concentration, bottom-strand concentration, template concentration, cycle number, and annealing temperature. For AccuStart HiFi reactions, the standard aPCR reaction was set using 1x AccuStart HiFi buffer (60 mM Tris-SO_4_ pH 8.9 at 25 °C, 18 mM (NH_4_)_2_SO_4_) with 2 mM MgSO_4_, 200 µM dNTPs, 1 µM forward primer, 20 nM reverse primer, and 30 ng template DNA (single-stranded M13mp18 genomic DNA from NEB or synthetic double stranded gBlock from Integrated DNA Technologies, Gibson assembled dsDNA^33^, or Phusion generated and purified double stranded DNA template), brought to 50 µL with nuclease-free water (IDT), with 0.22 µL AccuStart HiFi enzyme added to begin synthesis. Thermocycled reactions were initiated for 1 min at 94 °C, and thereafter cycled 30 times for 20 s at 94 °C, 30 s at 55 °C, and 90 s per kilobase at 68 °C. Variations in cycling parameters were performed about this reaction, with 20 µL reactions combined with 4 µL 6x loading buffer ran on 1% low-melt agarose cast with 1x SybrSafe and ran for 1 hour at 90 V. Gels were visualized with a blue-light illumination and band intensities were quantitated by integration using ImageJ^31,32^.

LongAmp reactions were similar in setup to the AccuStart HiFi. The standard reaction was in 1x LongAmp buffer (60 mM Tris-SO_4_, 20 mM (NH_4_)_2_SO_4_, 2 mM MgSO_4_, 3% glycerol, 0.06% IGEPAL CA-630, 0.05% Tween 20, pH 9.1 at 25 °C), 300 µM dNTPs, 1 µM forward primer, 20 nM reverse primer, 0.5–5 ng template DNA (lambda phage genomic DNA) and 2 µL LongAmp *Taq* DNA polymerase mix and brought to 50 µL. Thermocycled reactions were initiated for 30 s at 94 °C and cycled 30 times for 20 s at 94 °C, 45 s at 56.5 °C, and 90 s per kilobase at 65 °C. Parameter optimization was carried out by varying this general reaction. Samples were run on a 0.8% low-melt agarose gel stained with either ethidium bromide or SybrSafe for 1–1.5 hours at 100 V. Gels were visualized with UV or blue light and band densities were quantified by integration with ImageJ^31,32^.

Titrations of reaction component concentrations including magnesium, template, and reverse primer concentrations, and varying the number of cycles were carried out for both HiFi and LongAmp reactions for 1,000 nt and 3,281 nt ssDNA products. Reaction conditions and associated band intensities are presented in External Table 1. Purified ssDNA quantitated by UV absorbance at 260 nm wavelength was titrated on an agarose gel, and band intensities were measured using ImageJ to generate a standard intensity curve, which was then used to quantitate ssDNA production from a triplicate of aPCR reactions.

### ssDNA purification

ZymoClean Gel DNA Recovery Kit (Zymo Research) was used for ssDNA gel purification. Briefly, after excising the gel band containing the ssDNA product with a clean razor blade, 750 µL (3 volumes) of the provided binding buffer were added to the excised gel, and left to melt in an incubator at 45 °C for 10 min. The melted agarose gel solution was transferred to the silica-based spin columns and mounted on a collection tube, and centrifuged for 60 s at 11,000 RPM. After discarding the flow-through, 250 µL of ethanol-based DNA wash buffer were used to wash the column twice by centrifuging for 60 s at 11,000 RPM and discarding the flow-through each time. The ssDNA was recovered with 6–15 µL of elution buffer, after centrifugation for 60 s at 11,000 RPM. The concentration of recovered ssDNA was measured using a NanoDrop™ 2000 UV-Vis Spectrophotometer (Thermo Fisher Scientific Inc.). Final purified ssDNA was verified by Sanger sequencing from the 3′ end.

### Modified dNTPs incorporated into long ssDNA

Modified dNTPs (Alpha Thiol dATP, Alpha Thiol dCTP, Alpha Thiol dTTP, Alpha Thiol DGTP, Biotin-16-dCTP, dUTP, Cyanine 5-AA-CTP) as well as non-modified dNTPs (dATP, dCTP, dGTP, dTTP) were purchased from Trilink Biotechnologies, LLC. The modified dNTPs were incorporated by replacing the corresponding non-modified dNTPs at the indicated percentages.

dUTP was incorporated into the ssDNA by replacing dTTP with equal amounts of dUTP, up to 100% replacement using the linear asymmetric production technique described above. A gene encoding mCherry fluorescent protein was Phusion amplified using standard dNTPs (200 µM dTTP/0 µM dUTP) and gel and column purified. 200 ng of this template was used in the standard 50 µL AccuStart HiFi condition with the absence of a reverse primer. Uracil-containing ssDNA was initiated with the forward primer containing dUTP instead of dTTP but still at 1 µM final reaction concentration. dUTP:dTTP concentrations tested were 180:20 µM for 90%, 195:10 µM for 95%, and 200:0 µM for 100% dUTP.

To test biotinylation of the ssDNA with interest for further bioconjugation or pull-down assays, Biotin-16-dCTP were added to the dNTPs mix up to 100% replacement of the canonical dCTP.

To test backbone-modified nucleic acid incorporation with specific application in polymer stability, Alpha Thiol dNTPs were added. A mix of native and Alpha Thiol dNTPs were prepared at 10 mM ranging from 10 to 100% of Alpha Thiol dNTPs.

To test bulky, base-modified nucleic acid incorporation with specific application in fluorescent tracking, Cy5-dCTP was added. Cy5-dCTP was used at concentrations ranging from 0.5 to 10%, replacing the natural dCTP nucleotides. The incorporation of Cy5-dNTPs was monitored by in-gel fluorescence using a GE Typhoon™ FLA 7000 imager, monitored at λ_ex_: 473; emission filter Y520 for ethidium bromide and λ_ex_: 635 nm; emission filter R670 for Cy5. Gel purified ssDNA product was monitored by fluorescence measurements (λ_ex_: 620; λ_em_: 640–740) using a Horiba FluoroMax™-4.

### Folding scaffolded DNA origami nanoparticles

A DNA-scaffolded pentagonal bipyramid and tetrahedron was folded using 2,000 nt and 1,087 nt scaffolds, respectively, and using staples from DAEDALUS software and following the protocol described previously^11^, but with fluorescent Cy5-labeled scaffold. Briefly, annealing the nanostructure was performed in 50 µL of buffer Tris-acetate EDTA-MgCl_2_ buffer (40 mM Tris, 20 mM acetic acid, 2 mM EDTA, 12 mM MgCl_2_, pH 8.0) with DNA scaffold at 40 nM concentration and a 10x molar excess of staple strands. The program used for annealing was the following: 95 °C for 5 min, 80–75 °C at 1 °C per 5 min, 75–30 °C at 1 °C per 15 min, and 30–25 °C at 1 °C per 10 min. The folded structure was run on a 2% agarose gel pre-stained with ethidium bromide. Fluorescent scaffold and nanoparticles were visualized using a GE Typhoon™ FLA 7000 imager, monitored at λ_ex_: 473; emission filter Y520 for ethidium bromide and λ_ex_: 635 nm; emission filter R670 for Cy5.

### Data availability

All data will be available upon request to the corresponding authors. The plasmid containing the 1,087 nt Cv3 insert is available from Addgene (plasmid #99351).

### Code availability

aPrime source code is available under the GPL v2 license at GitHub (https://github.com/lcbb/aPrime). The application is also accessible on the web at http://daedalus-dna-origami.org/aPrime.

## Electronic supplementary material

Supplementary Information

Supplementary Tables
