# Supplementary material for: In vitro synthesis of gene-length single-stranded DNA
Source: Sci Rep. 2018 Apr 25;8:6548. doi: 10.1038/s41598-018-24677-5 (PMC5916881; doi:10.1038/s41598-018-24677-5)
Supplement: Supplementary file 1 — Supplementary Information [file 41598_2018_24677_MOESM1_ESM.docx]

**Supplementary information**

***In vitro* synthesis of gene-length single-stranded DNA**

Rémi Veneziano^1†^*, Tyson R. Shepherd^1†^, Sakul Ratanalert^1,2^, Leila Bellou^1^, Chaoqun Tao^1^, Mark Bathe^1^*

^1^Department of Biological Engineering, Massachusetts Institute of Technology, Cambridge, MA 02139, USA

^2^Department of Chemical Engineering, Massachusetts Institute of Technology, Cambridge, MA 02139, USA

*To whom correspondence should be addressed: [mark.bathe@mit.edu](mailto:mark.bathe@mit.edu) and rvenezia@mit.edu

^†^These authors contributed equally to this work.

**SUPPLEMENTARY MATERIALS AND METHODS**

**ssDNA purification**

Four commercial kits for ssDNA gel purification were evaluated for efficiency: ZymoClean Gel DNA Recovery Kit (Zymo Research), ZymoClean Gel RNA Recovery Kit (Zymo Research), NucleoSpin Gel and PCR Clean-up Kit (Macherey-Nagel), and MicroElute Gel Extraction Kit (Omega bio-tek). The kits were applied following protocols provided by the respective vendors. Briefly, after excising the gel band containing the ssDNA product with a clean razor blade, 750 µL (3 volumes) of the provided binding buffer were added to the excised gel, and left to melt in an incubator at 45°C for 10 min. The melted agarose gel solution was transferred to the silica-based spin columns and mounted on a collection tube, and centrifuged for 60 s at 11,500 x g. After discarding the flow-through, 250 µL of ethanol-based DNA wash buffer were used to wash the column twice by centrifuging for 60 s at 11,500 x g and discarding the flow-through each time. The ssDNA was recovered with 6–15 µL of elution buffer, after centrifugation for 60 s at 11,500 x g. The concentration of recovered ssDNA was measured using a NanoDrop™ 2000 UV-Vis Spectrophotometer (Thermo Fisher Scientific Inc.). Final purified ssDNA was verified by Sanger sequencing from the 3’ end.

The Quantum Prep Freeze ‘N Squeeze™ DNA Gel Extraction Spin Columns (Bio-Rad Laboratories, Inc.) were tested as an alternative method for ssDNA purification. The excised gel portion containing ssDNA was crushed into pieces, and placed in the spin column to be frozen for 5–15 minutes at -20°C before centrifugation at 16,000 x g for 5–20 minutes at room temperature, until the totality of the gel has been filtered. The ssDNA was then concentrated using ethanol precipitation, dried at 37°C for 1 hour, and resuspended in Tris-buffer. The flow-through solution contains the purified single-strand DNA, and the concentration was measured using a NanoDrop 2000 UV-Vis Spectrophotometer.

Electro-elution: The excised gel portion containing ssDNA was placed in a regenerated cellulose dialysis bag 3,000 MWCO (Sigma) with 300 µL of 1X Tris-acetate-EDTA (TAE) buffer. The bag was immersed in an electrophoresis tank filled with 1X TAE Buffer, and an electric current passed through the bag at 100 V for 30 min. The ssDNA electro-eluted out of the gel was collected and concentrated using ethanol precipitation, dried at 37°C for 1 hour, and resuspended in Tris-acetate buffer. The final concentration was measured using a NanoDrop™ 2000 UV-Vis Spectrophotometer.

**Linear asymmetric ssDNA production**

A 1,087 nt *de novo* synthetic DNA sequence (“Cv3”) was generated using a conversion of digital binary ASCII text data to a sequence of nucleotides, with flanking primers generated to satisfy the rules presented above. The sequence was flanked with descriptive barcode sequences. Further, the synthetic sequence was chosen to contain no biologically active sequences (i.e. long open reading frames, promoters, or ribosome binding sites) and to have no repeated or self-complementary sequences longer than 7 nucleotides, and no homopolymer repeats of longer than 4 nucleotides. The sequence was then flanked by PstI sites (CTGCAG) on the 5’ and 3’ ends and synthesized in gBlock format (IDT). The gBlock DNA was amplified using Phusion, purified by Qiagen MiniPrep kit, digested with PstI-HF (NEB), and gel purified. The cloning vector pUC19 was digested with PstI-HF and treated with shrimp alkaline phosphatase and gel purified. The digested insert was ligated to the digested pUC19 (40 ng: 30 ng ratio in 20 µL) using T4 DNA ligase overnight at room temperature in 1X ligase buffer. A 1 µL aliquot of the ligation was transformed in 20 µL DH5α chemically competent cells (NEB) by following the manufacturer’s protocol and plated to LA media supplemented with 100 µg/µL ampicillin and incubated overnight at 37°C. Individual colonies were cultured and DNA was purified by use of a Qiagen MiniPrep spin kit. DNA was digested with PstI and additionally tested by PCR and Sanger sequencing to validate the correct insert.

Double-stranded DNA template for asymmetric production was generated by traditional symmetric PCR with Phusion polymerase (ThermoFisher) with 1X HF buffer, 200 µM dNTPs, 250 nM forward and reverse primers (the same sequences used for the asymmetric reactions), 1–10 ng of template DNA, and amplified by 30 cycles using the annealing temperature recommended by the manufacturers’ calculations (63°C). Alternatively, the dsDNA template was generated by digestion from the pUC19 clone directly. 2 µg of plasmid DNA was incubated with PstI-HF. In each case, the reactions were run on 1% low-melt agarose and the template band was extracted from the gel and purified using the ZymoClean Gel DNA Recovery Kit (Zymo Research) and then column purified a second time using a Qiagen spin column purification (Qiagen Miniprep kit). Subsequent ssDNA production was set up with 1X AccuStart HiFi buffer, 2 mM MgSO_4_, 1 µM forward primer, and 100–300 ng of dsDNA purified template in 50 µL total reaction. Thermocycling was performed for 20 cycles at 55°C, with all other conditions identical to the aPCR protocol.

**Production of ssDNA from Gibson-assembled gBlocks**

Two gBlocks (see sequences in SI External Table) were designed with 40 nt overlapping regions and were assembled following the protocol provided by the vendor (NEB). Briefly, 0.2 pmoles of the two gBlock fragments were mixed with 10 μL of 2X Gibson assembly® Master Mix and the reaction volume was adjusted to 20 μL with PCR grade water, and incubated at 50°C for 1 hour. The assembled product (without any purification) was used directly to perform aPCR experiments (2 μL of assembled product for 50 μL of aPCR reaction).

**Table S1. Commercial enzymes evaluated for aPCR optimization of ssDNA amplification.**

| **Enzyme Type** | **Taq** | **Taq** | **Taq** | **Taq** | **Taq** | **Taq** |
| --- | --- | --- | --- | --- | --- | --- |
| **Enzyme Name** | Accustart™ | Accustart™ HiFi | Accustart™II | AccuPrime™ | GoTaq® | DreamTaq™ |
| **Buffer (1x)** | 20 mM Tris-HCl, 50 mM KCl, 2 mM MgCl_2_, pH 8.4 | 60 mM Tris-SO_4_, 18 mM, (NH_4_)_2_SO_4_, 2 mM MgSO_4_, pH 8.9 | PCR Buffer II, 2 mM MgCl_2_ | AccuPrime™ PCR buffer: 20 mM Tris-HCl, 50 mM KCl, 1.5 mM MgCl_2_, pH 8.4, 200 uM dGTP, 200 uM dATP, 200 uM dATP, 200 uM dCTP, thermostable AccuPrime™ protein, 1% glycerol | Colorless GoTaq™ reaction buffer: 1.5 mM MgCl_2_, pH 8.5 | DreamTaq buffer: 2 mM MgCl_2_ |
| **Provider** | Quantabio | Quantabio | Quantabio | Thermo-Fisher | Promega | Thermo-Fisher |
|  |  |  |  |  |  |  |
| **Enzyme Type** | **Phusion®** | **Platinum®** | **Q5®** | **Tth** | **Deep vent®** | **Deep Vent®** |
| **Enzyme Name** | Phusion® | Platinum™ SuperFi™ | Q5® hot start HiFi | Tth | Deep Vent® | Deep Vent® (exo-) |
| **Buffer (1x)** | Phusion® HF buffer. 2 mM MgCl_2_ | SuperFi™ buffer. 1.5 mM MgCl_2_ | Q5® reaction buffer: 2 mM MgCl_2_ | 10 mM Tris-HCl, 50 mM KCl, 1.5 mM MgCl_2_, pH 8.6 | ThermoPol reaction buffer: 20 mM Tris-HCl, 10 mM (NH_4_)_2_SO_4_, 10 mM KCl, 2 mM MgSO_4_, 0.1% Triton X-100, pH 8.8 | ThermoPol reaction buffer: 20 mM Tris-HCl, 10 mM (NH_4_)_2_SO_4_, 10 mM KCl, 2 mM MgSO_4_, 0.1% Triton X-100, pH 8.8 |
| **Provider** | NEB | Thermo-Fisher | NEB | Affymetrix | NEB | NEB |


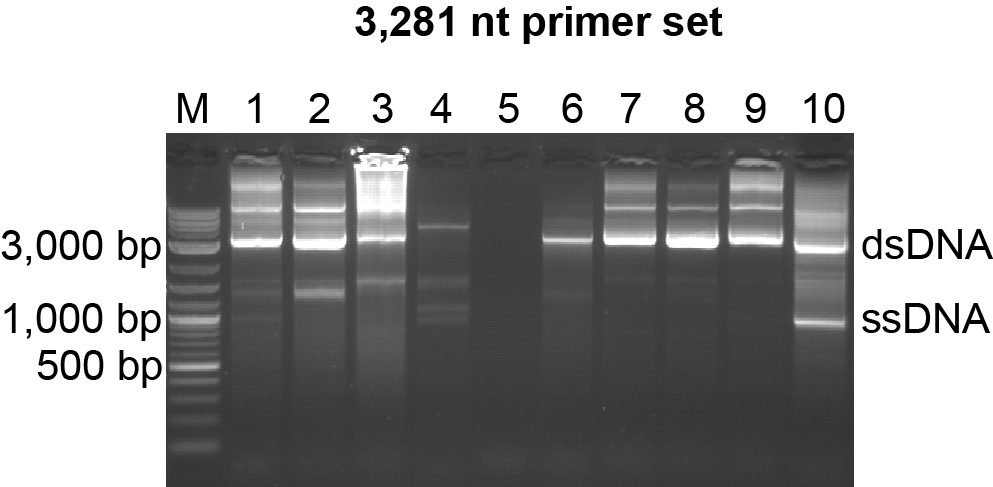


**Figure S1. Enzyme selection for the amplification of a 3,281 nt fragment from the M13mp18 ssDNA template.** 1. Accustart; 2. Accustart HiFi; 3. Accustart II; 4. AccuPrime; 5. GoTaq; 6. DreamTaq; 7. Phusion; 8. Platinum SuperFi; 9. Q5; 10. Tth polymerase. PCR samples were run on a 1% agarose gel prestained with EtBr.


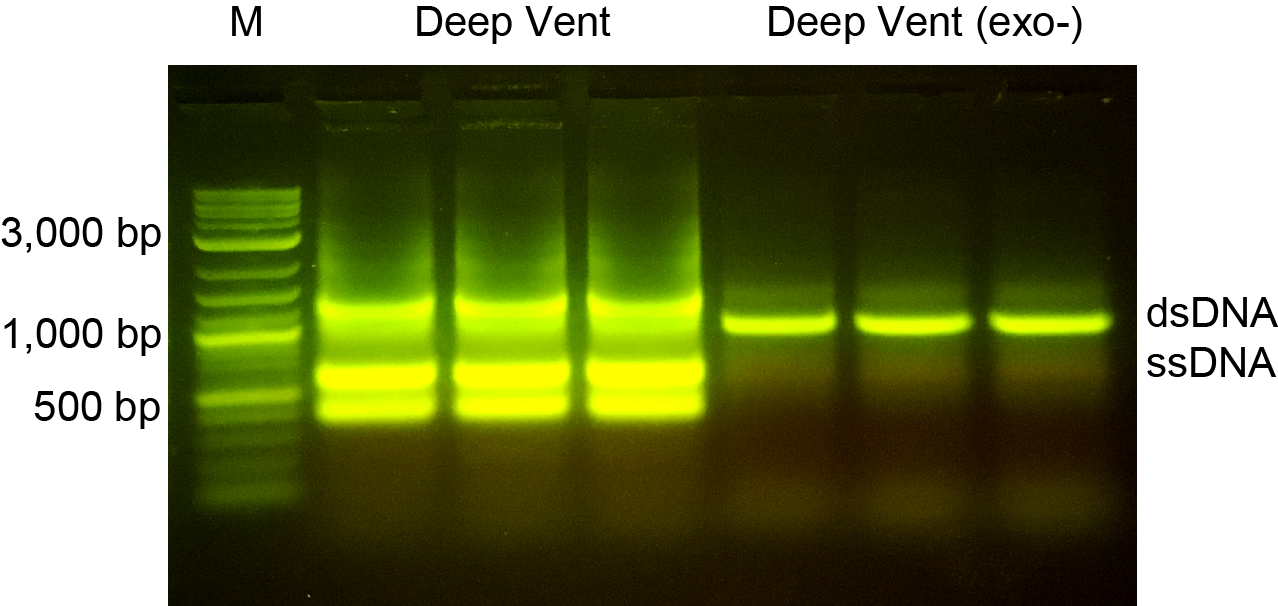


**Figure S2. Impact of exonuclease activity effect on 1,000 nt ssDNA production.** Comparison of the NEB DNA polymerases Deep Vent® and the Deep Vent (exo-)® lacking the 3’ to 5’ exonuclease activity for amplification of a 1,000 nt ssDNA fragment using M13mp18 ssDNA as template using the optimized aPCR protocol. aPCR products were run on a 1% low melt agarose gel electrophoresis prestained with SybrSafe.


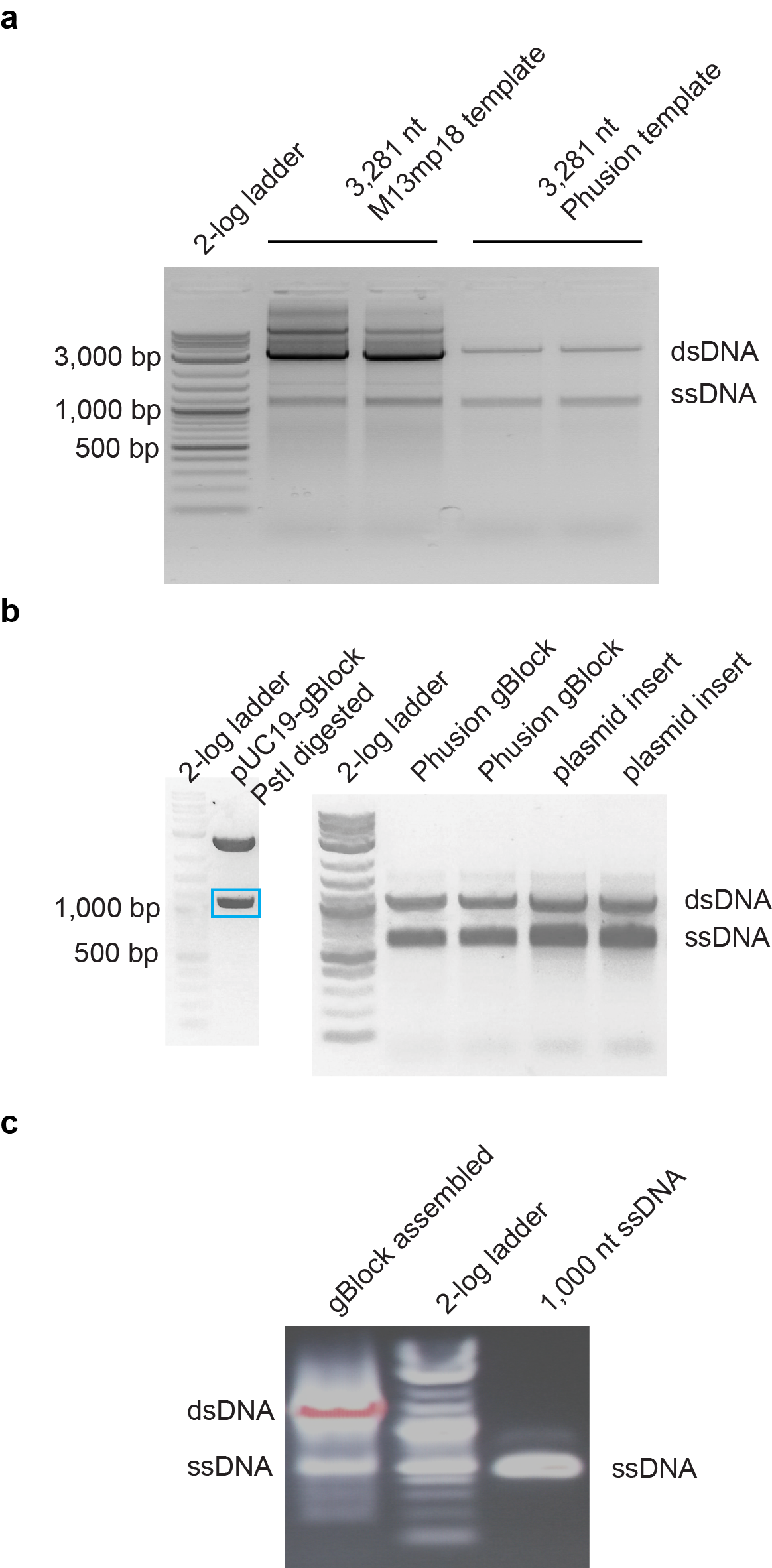


**Figure S3. Asymmetric amplification from pre-synthesized template. a.** Amplification of the 3,281 nt fragment with aPCR using M13mp18 ssDNA circular template, compared with Phusion amplified 3,281 dsDNA template without the reverse primer for direct production of ssDNA. **b.** Amplification of 1,087 nt synthetic ssDNA sequence using templates derived from *Escherichia coli* cloned plasmid or from Phusion-amplified templates. Left, synthetic sequence cloned to pUC19 using PstI can be gel purified and used as a template for ssDNA production. Right, Phusion-amplified and bacterially-produced templates were used to produce ssDNA without a reverse primer. **c.** ssDNA amplification with aPCR of a 1,391 nt template assembled using Gibson assembly protocol (33).**Table S2. Primers generated by the primer design algorithm applied to generate the data shown in Figure S3.**

| **Size (nt)** | **Forward primer** | **Reverse primer** |
| --- | --- | --- |
| 1,087 (gBlock) | GTCGTCGTCCCCTCAAACTC | GCTGAAAAGGTGGCATCAAT |


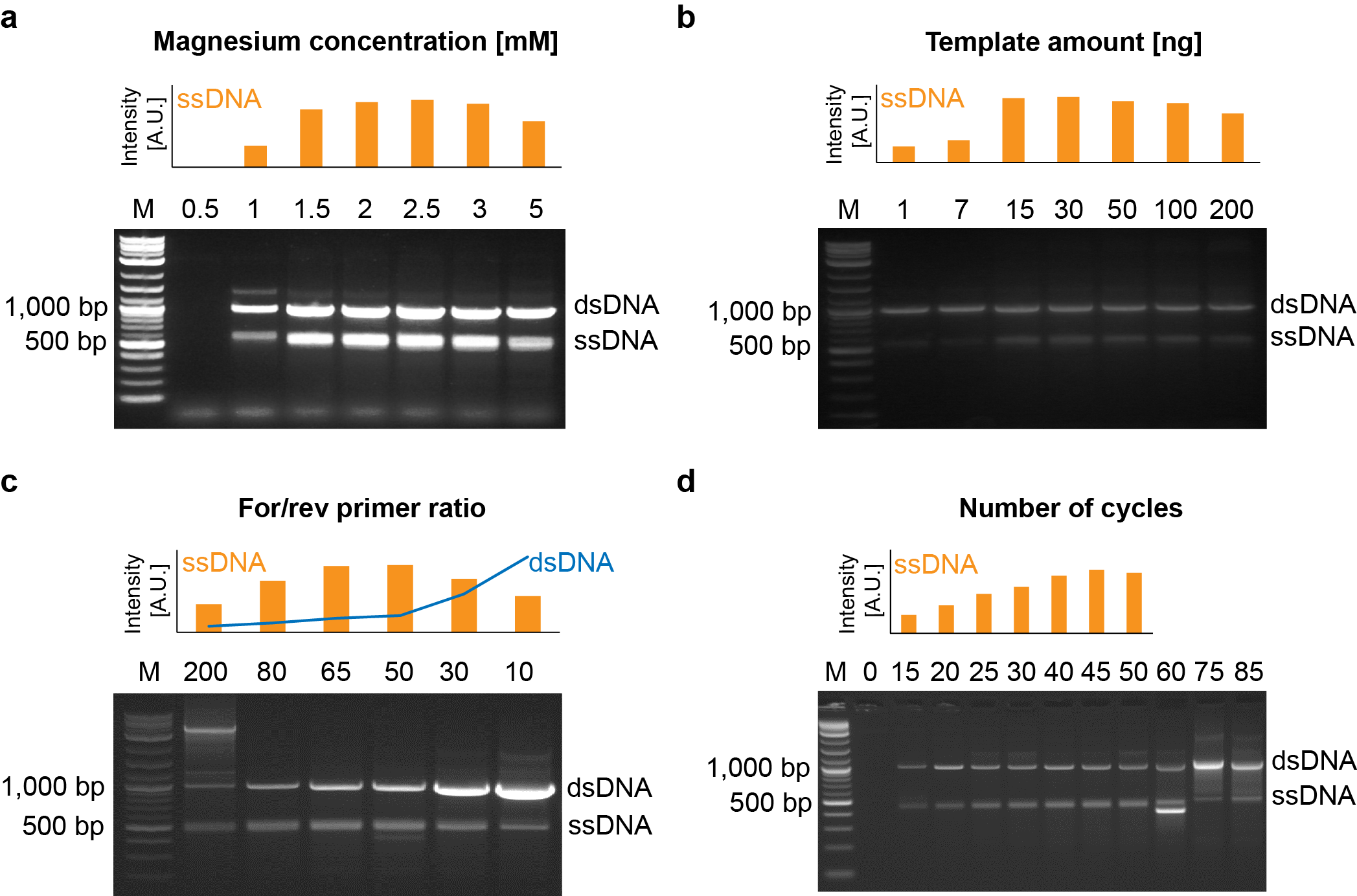


**Figure S4. aPCR components and conditions for maximizing ssDNA yield with M13mp18 ssDNA as template.** **a.** Titration of magnesium concentration for the amplification of a 1,000 nt ssDNA fragment. **b.** Titration of M13mp18 circular ssDNA template quantity used for the amplification of a 1,000 nt ssDNA fragment. **c.** Test of different ratios of the forward to reverse primer for the amplification of a 1,000 nt ssDNA fragment. **d.** Optimization of the number of cycles of the aPCR protocol for the amplification of a 1,000 nt ssDNA fragment. aPCR products were run on a 1% low melt agarose gel prestained with ethidium bromide and the band intensity was determined using ImageJ software (31, 32).


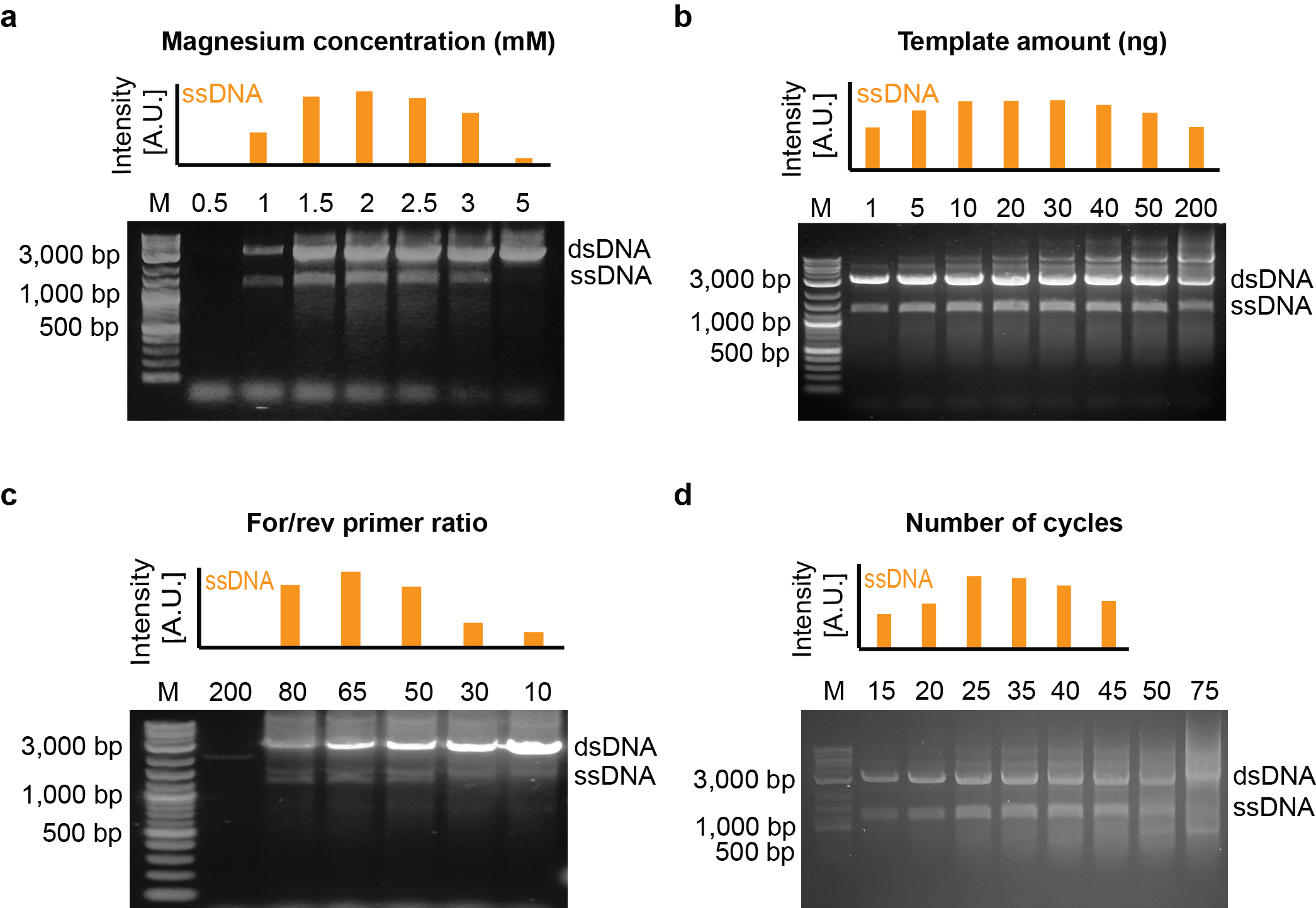


**Figure S5. Optimization of aPCR components and conditions for maximizing ssDNA yield using M13mp18 ssDNA as template.** Optimization of **a**. the magnesium concentration, **b.** the template quantity used, **c.** the ratio of forward and reverse primers**,** and **d.** the number of cycles for the amplification of the 3,281 nt fragment with the Quantabio Accustart HiFi polymerase. The bar graphs represent the intensity of the ssDNA and dsDNA bands measured by ImageJ (31, 32) of the gels shown.


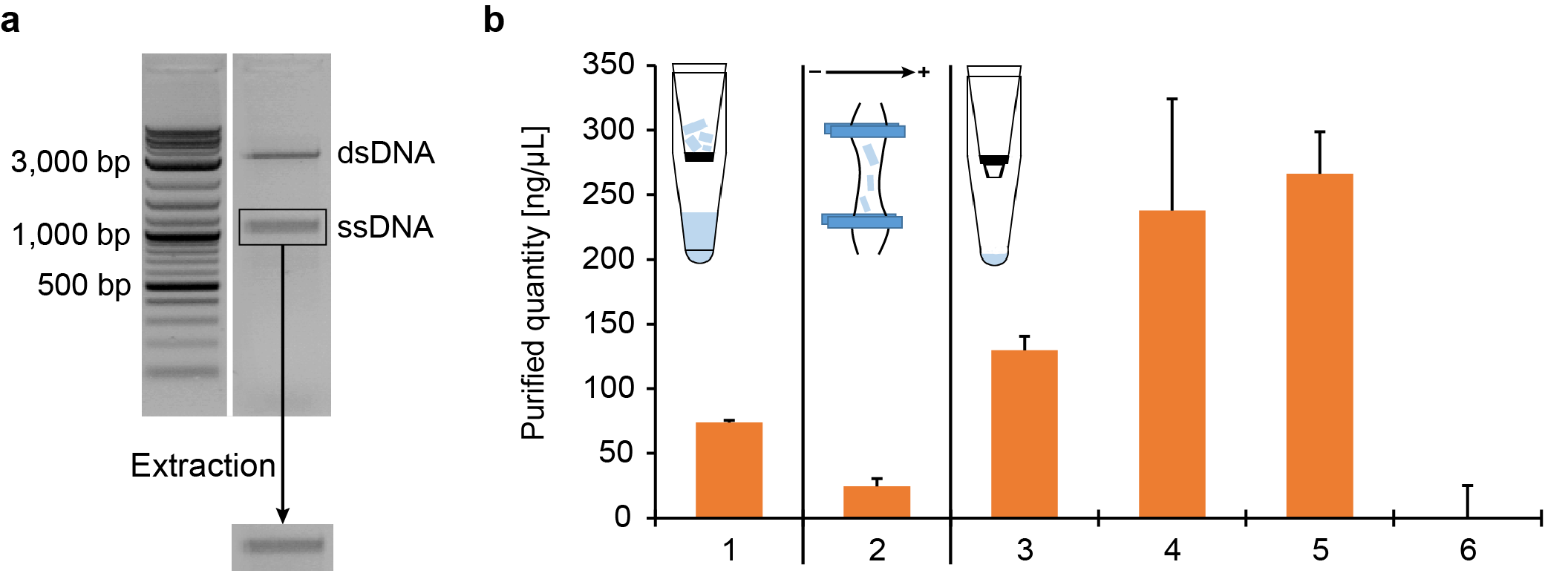


**Figure S6. Purification of ssDNA.** **a.** aPCR products are run on agarose gel and the ssDNA band is extracted before purification. The gel presented here is used for illustration. **b.** Quantification with NanoDrop™ of ssDNA production after purification using six different techniques. 1: Freeze ‘N Squeeze kit; 2: Electro-elution with 12 kDa dialysis membrane; 3: MicroElute Gel Extraction kit; 4: PCR clean-up kit; 5: ZymoClean Gel DNA Recovery Kit; 6: ZymoClean Gel RNA Recovery kit. The bar heights represent the average purified quantity of at least three experimental replicates (12 tubes of 50 μL aPCR reaction for each replicate) for each kit with standard deviation shown as error bars.


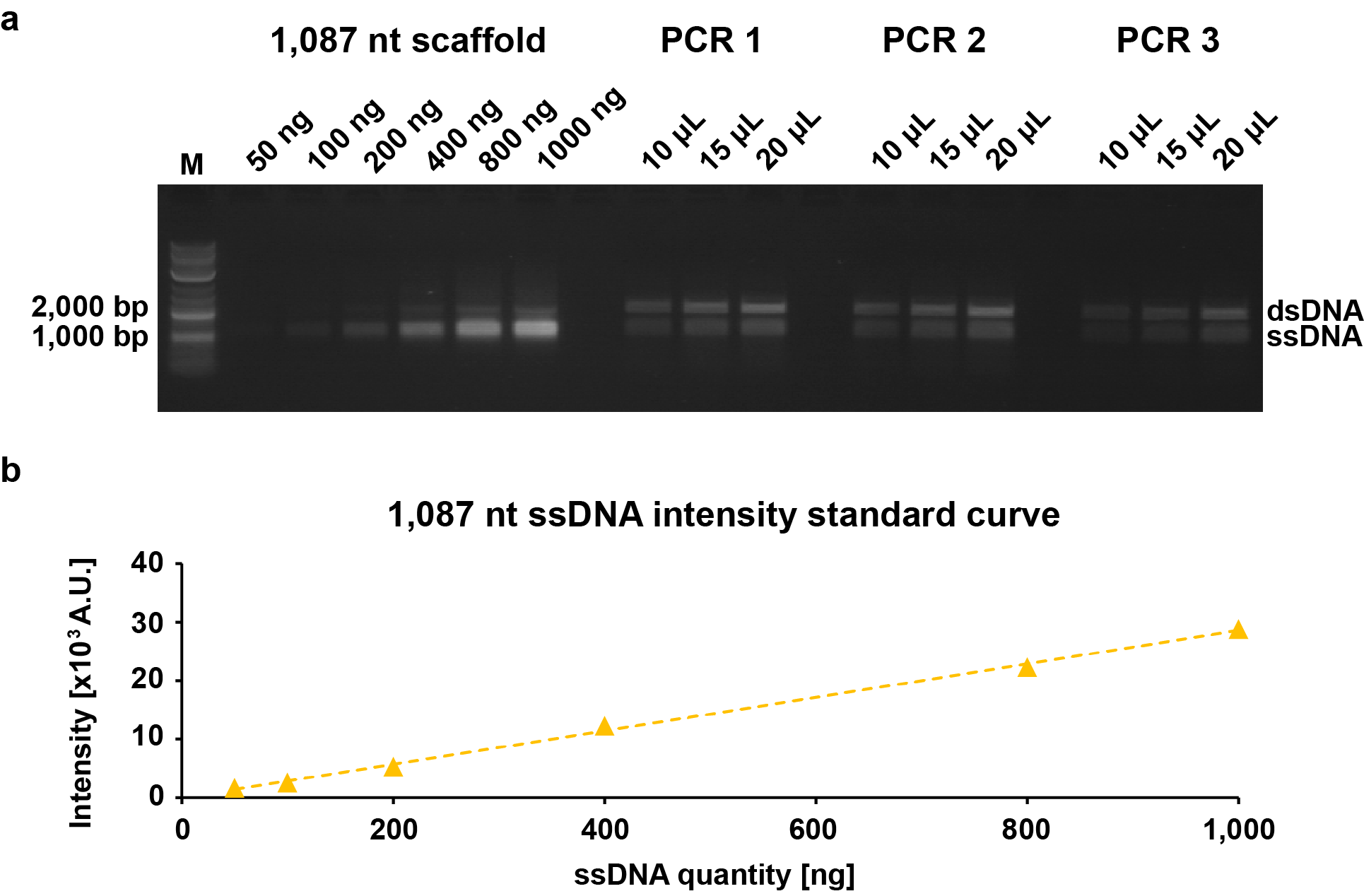


**Figure S7. Quantification of ssDNA produced by aPCR. a.** Increasing quantities of purified 1,087 nt ssDNA scaffold were loaded on a 1% agarose gel at the same time as three independent raw PCR reactions freshly prepared for production of 1,087 nt ssDNA. **b.** Average band intensities measured with ImageJ software (31,32) of increasing quantities of 1,087 nt ssDNA, which serve as a standard curve.


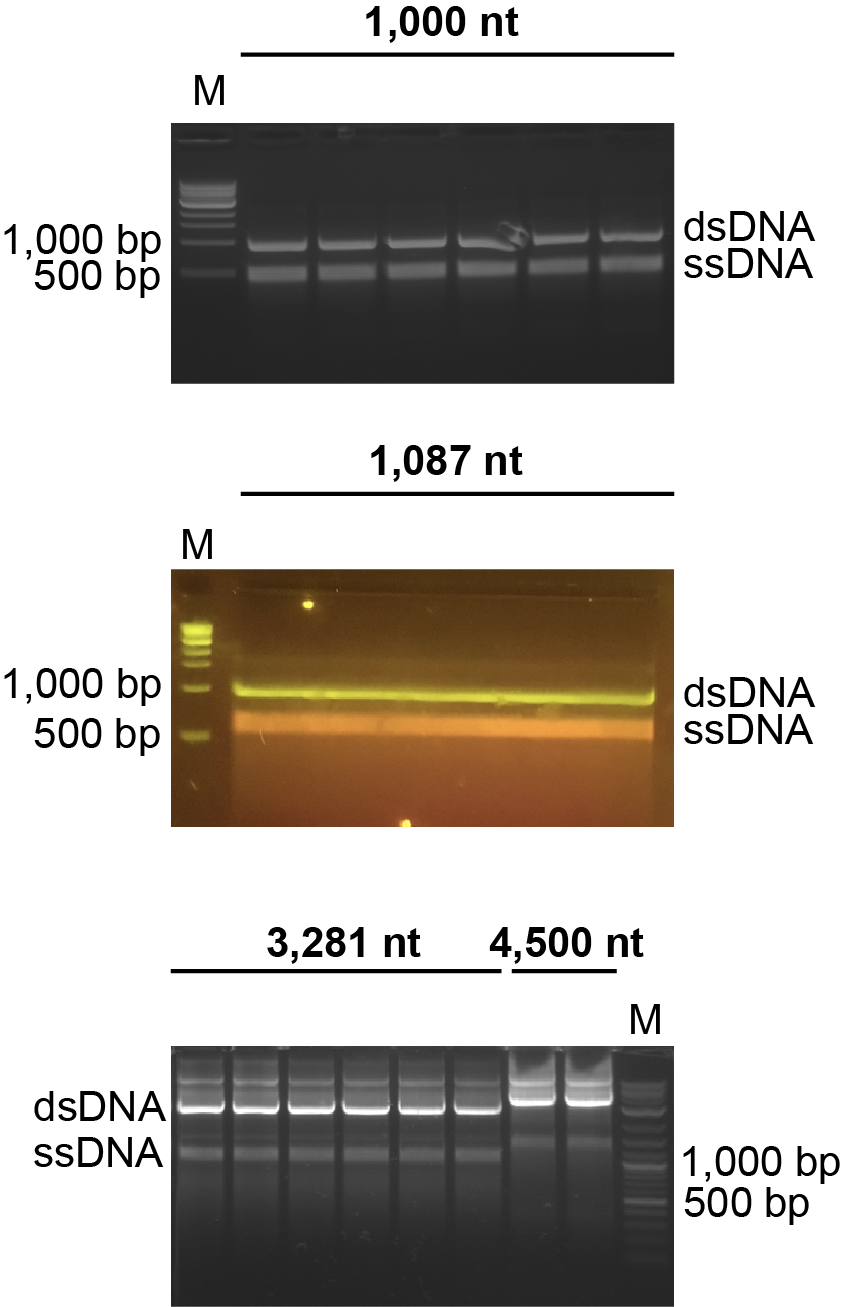


**Figure S8. Reproducibility of the ssDNA amplification with aPCR for various size.** Amplification of multiple replicates of the 1,000, the 1,087, the 3,281 and 4,500 nt fragments showing similar ssDNA production yield after gel electrophoresis using the M13mp18 ssDNA as template.


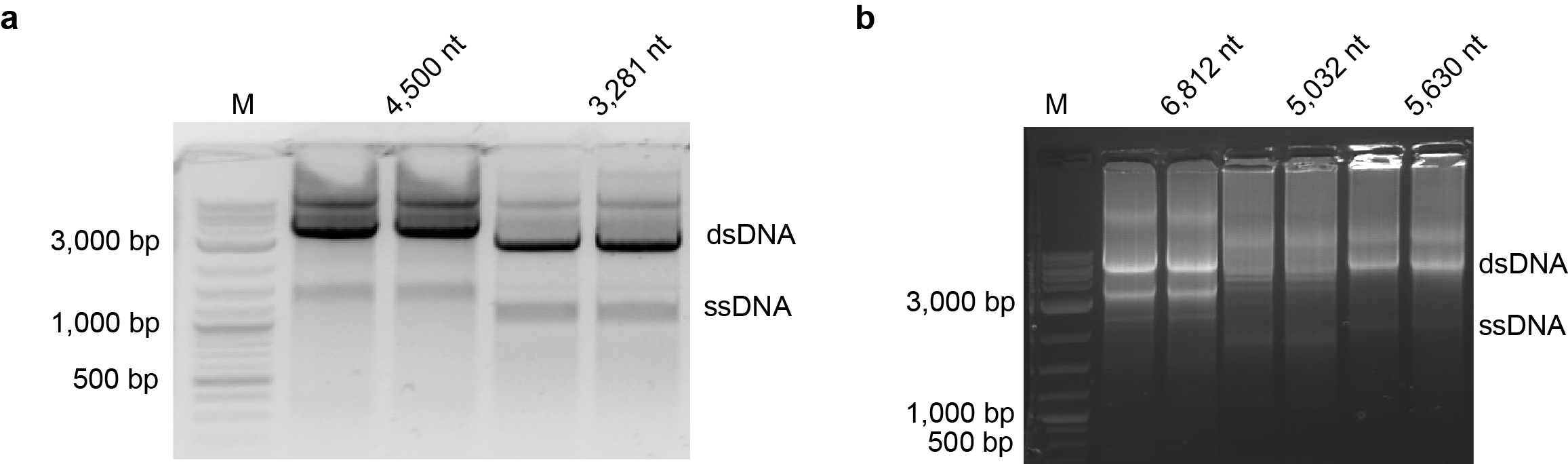


**Figure S9. Processivity of the Quantabio Accustart HiFi enzyme using the optimized aPCR protocol using the M13mp18 ssDNA as template. a.** Amplification of the 3,281 and 4,500 nt fragments and **b.** Amplification of the 5,032, the 5,630 nt, and the 6,812 nt fragments showing reduction of the ssDNA production for fragments longer than 6,000 nt.

**Table S3. Primers generated by the primer design algorithm applied to generate the data shown in Figure S9.**

| **Size (nt)** | **Forward primer** | **Reverse primer** |
| --- | --- | --- |
| 4,500 | TGTACTTTGTTTCGCGCTTG | AGGGAAGAAAGCGAAAGGAG |
| 5,032 | TGCCTCAACCTCCTGTCAAT | GCTGAAAAGGTGGCATCAAT |
| 5,630 | CTACCCTCGTTCCGATGCT | ATTAATGCCGGAGAGGGTAG |
| 6,812 | gcgacgatttacagaagcaa | GGCATTTTCGAGCCAGTAAT |


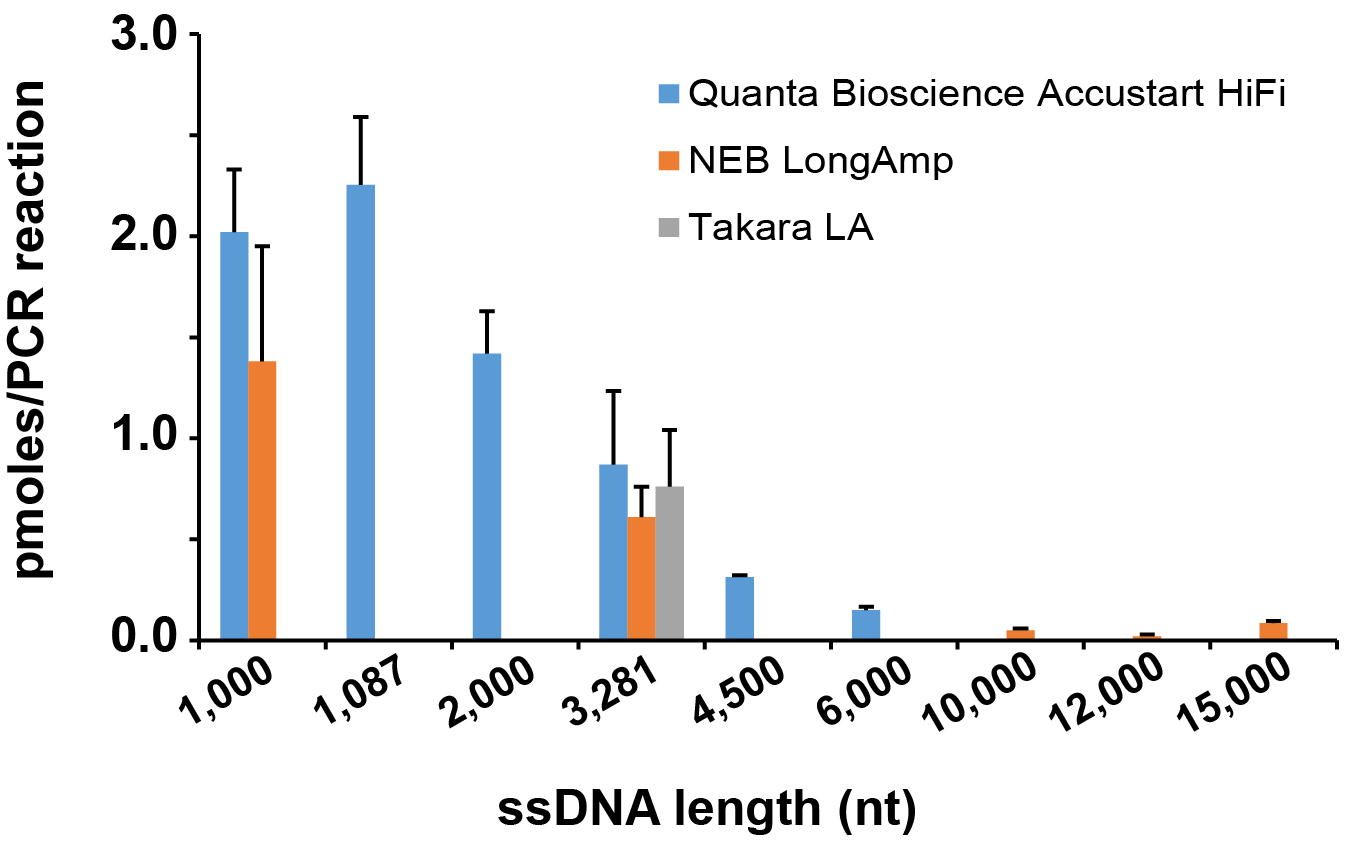


**Figure S10. Long ssDNA production with QuantaBio Accustart HiFi, NEB LongAmp, and Takara LA enzymes.** Bar height represents the average quantity of ssDNA produced per aPCR reaction from the replicates quantified in **External Table S2** (n=11 for 1,000 Accustart HiFi, n=2 for 1,000 LongAmp; n=4 for 1,087 Accustart HiFi; n=8 for 2,000 Accustart HiFi; n=11 for 3,281 Accustart HiFi, n=2 for 3,281 LongAmp, and n=2 for 3,281 LA; n=3 for 4,500 Accustart HiFi; n=2 for 6,000 Accustart HiFi; n=6 for 10,000 LongAmp; n=11 for 12,000 LongAmp; n=4 for 15,000 LongAmp). Error bars denote standard deviation.


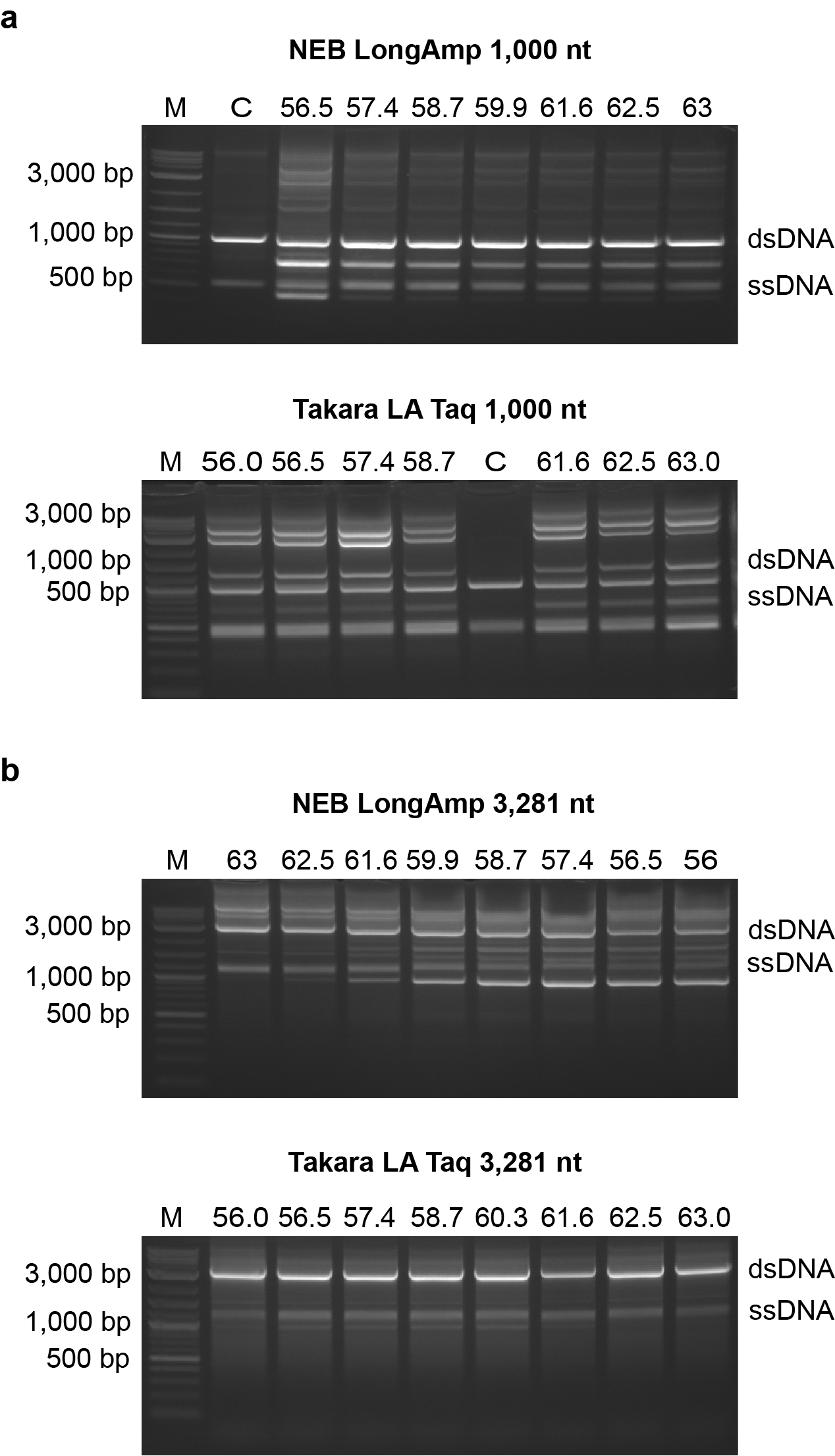


**Figure S11. Optimization of the hybridization temperature for amplification of ssDNA fragments with long amplification polymerases using M13mp18 ssDNA as template.** Amplification of **a.** 1,000 nt or **b.** 3,281 nt ssDNA fragments using different hybridization temperature for NEB LongAmp or Takara LA polymerases.

| **Enzyme Type** | **Taq** | **Taq** |
| --- | --- | --- |
| **Enzyme Name** | LongAmp Taq | LA Taq |
| **Buffer (1x)** | LongAmp *Taq* reaction buffer: 60 mM Tris-SO_4_, 20 mM (NH_4_)_2_SO_4_, 2 mM MgSO_4_, 3% glycerol, 0.06% IGEPAL CA-630, 0.05% Tween 20, pH 9.1 | LA PCR buffer II: 2.5 mM MgCl_2_ |
| **Provider** | NEB | Takara |

**Table S4. Commercial enzymes evaluated for amplification of long fragments (>3,000 nt) using aPCR.**


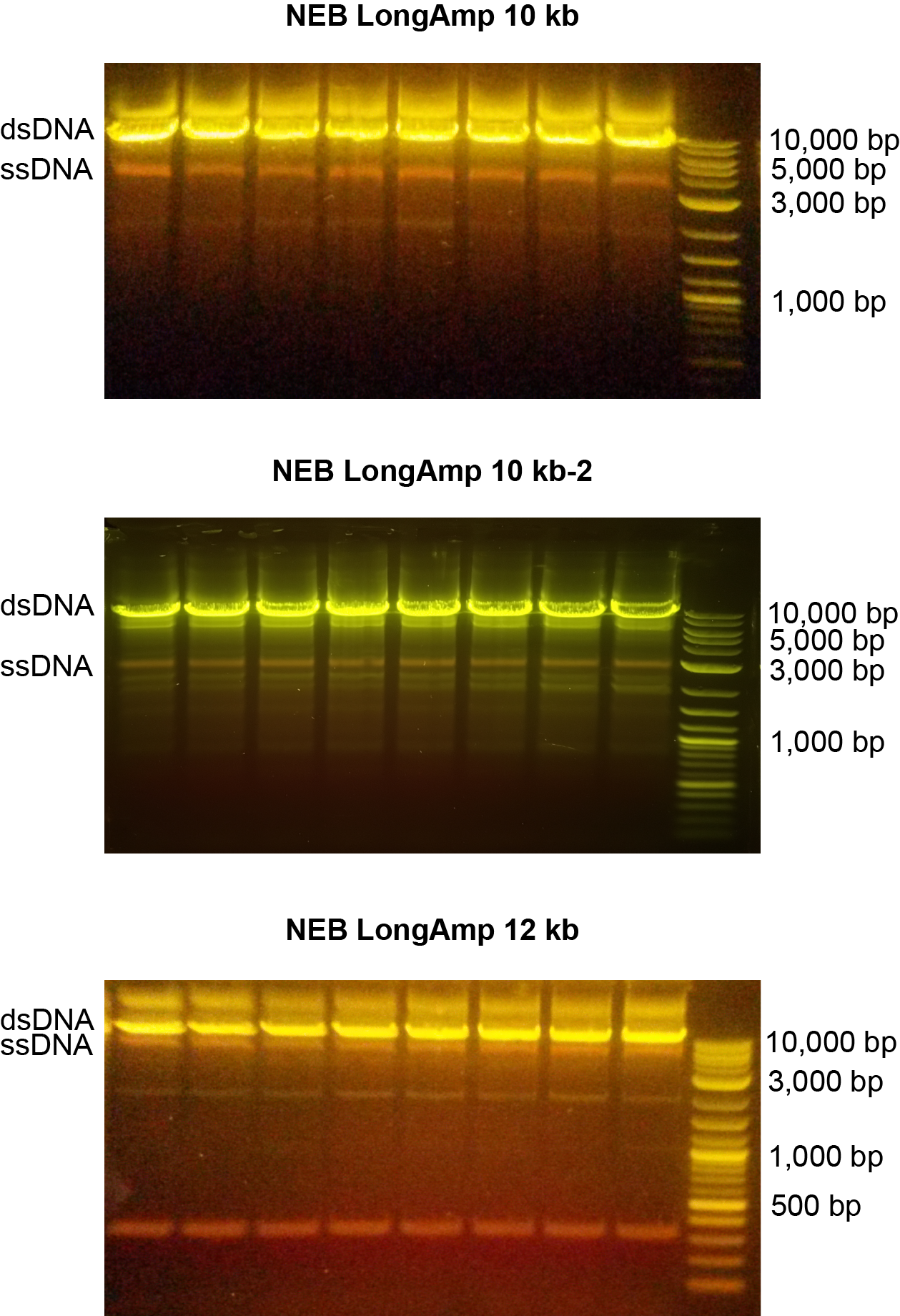


**Figure S12. Amplification of two 10 kb fragments and 12 kb ssDNA fragments using the NEB LongAmp polymerase and the Lambda phage dsDNA plasmid as template.** Each line represents one individual replicate (50 μL) of aPCR, showing the reproducibility of the experiment using the Lambda phage as template.


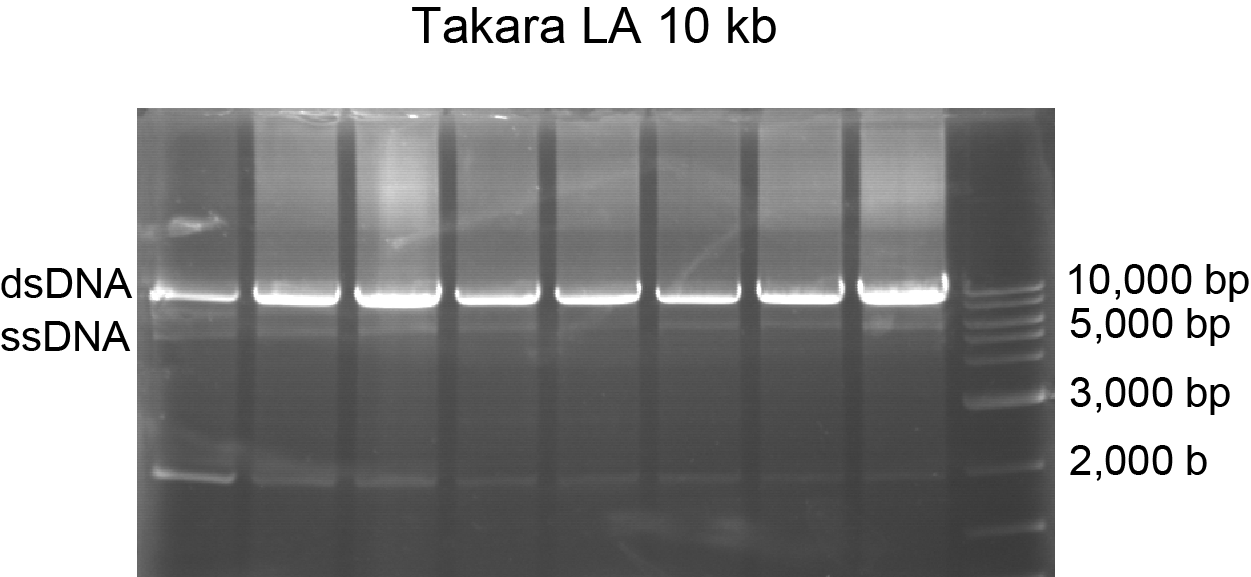


**Figure S13. Amplification of the 10 kb ssDNA fragment using the Takara LA Taq polymerase.** Lambda phage is use as template.


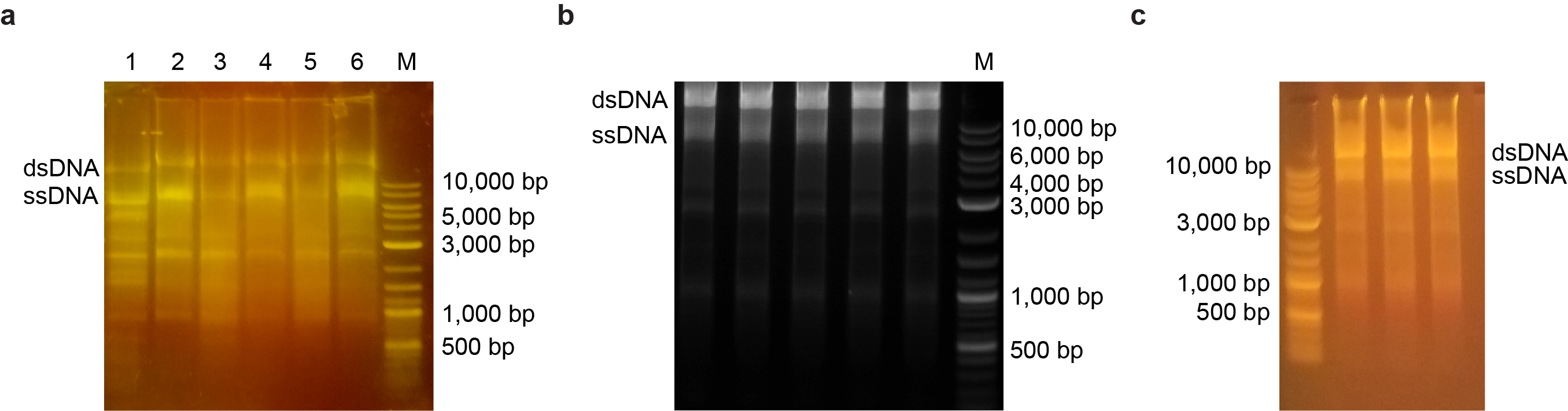


**Figure S14. Amplification of the 15 kb ssDNA fragment from Lambda phage dsDNA template. a.** Comparison of NEB LongAmp and LongAmp Hot Start with various quantity of Lambda phage dsDNA template. 1. LongAmp with 1 ng of template; 2. LongAmp Hot Start with 1 ng of template; 3. LongAmp with 5 ng of template; 4. LongAmp Hot Start with 5 ng of template; 5. LongAmp with 25 ng of template; 6. LongAmp Hot Start with 25 ng of template. **b. and c.** Amplification of 15 kb ssDNA with 5 ng of Lambda phage as template per reaction showing reproducibility of the aPCR production of ssDNA.


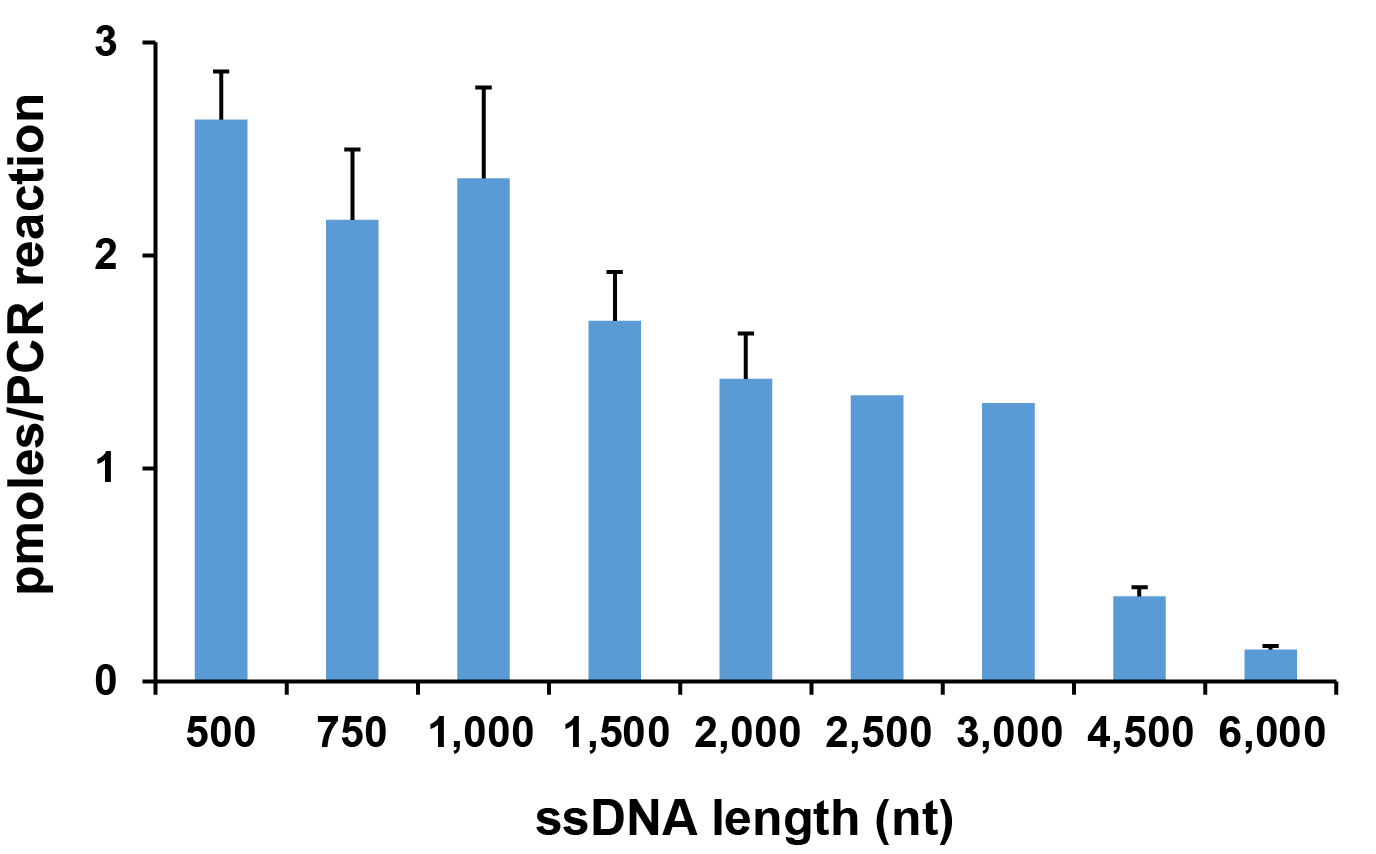


**Figure S15. Graph of pmoles per 50 µL aPCR reaction for products of different lengths.** Results are obtained by the combination and purification of three individual 50 μL PCR reactions. Error bars represent standard deviation from the average of the purification experiments quantified in **External Table S2** (n=2 for 500; n=3 for 750; n=11 for 1,000; n=8 for 1,500; n= 8 for 2,000; n=1 for 2,500; n=1 for 3,000; n=3 for 4,500; n=2 for 6,000).

**Table S5. Primers generated by the primer design algorithm and applied to generate the data shown in Figure 1d.**

| **Size (nt)** | **Forward primer** | **Reverse primer** |
| --- | --- | --- |
| 500 | GGACGCTATCCAGTCTAAACAT | GAAAGAGGACAGATGAACGGTG |
| 750 | CGCTTTCTTCCCTTCCTTTCT | GGCGATTAAGTTGGGTAACGC |
| 1000 | CTCGGTGGCCTCACTGATTAT | GCTGCAAGGCGATTAAGTTGG |
| 1500 | GGGCTTGCTATCCCTGAAAAT | GACTTGCGGGAGGTTTTGAAG |
| 2000 | GATGAGTGCGGTACTTGGTTTA | GCTTTGACGAGCACGTATAACG |
| 2500 | CACGGTCGGTATTTCAAACCA | CCTCTTCGCTATTACGCCAGC |
| 3000 | GCACTGACCCCGTTAAAACTTA | CAGATTCACCAGTCACACGACC |
| 4500 | TGTACTTTGTTTCGCGCTTG | AGGGAAGAAAGCGAAAGGAG |
| 6000 | GATGAGTGCGGTACTTGGTT | GAATGGAAAGCGCAGTCTCTG |

**
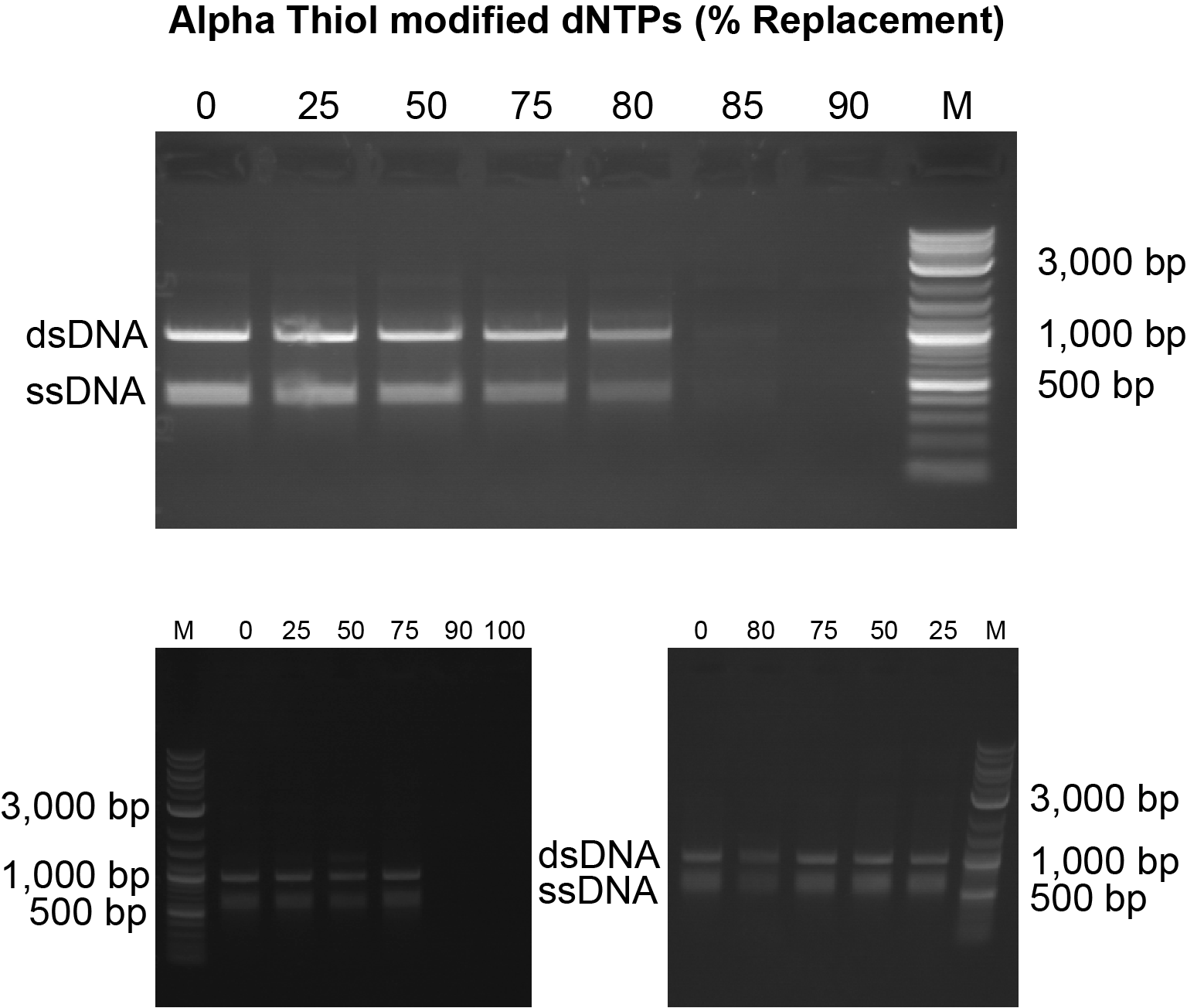
**

**Figure S16. Amplification of a 1,000 nt ssDNA fragment with increasing percent replacement of dNTPs with Alpha Thiol modified dNTPs, as indicated in the associated lane.**


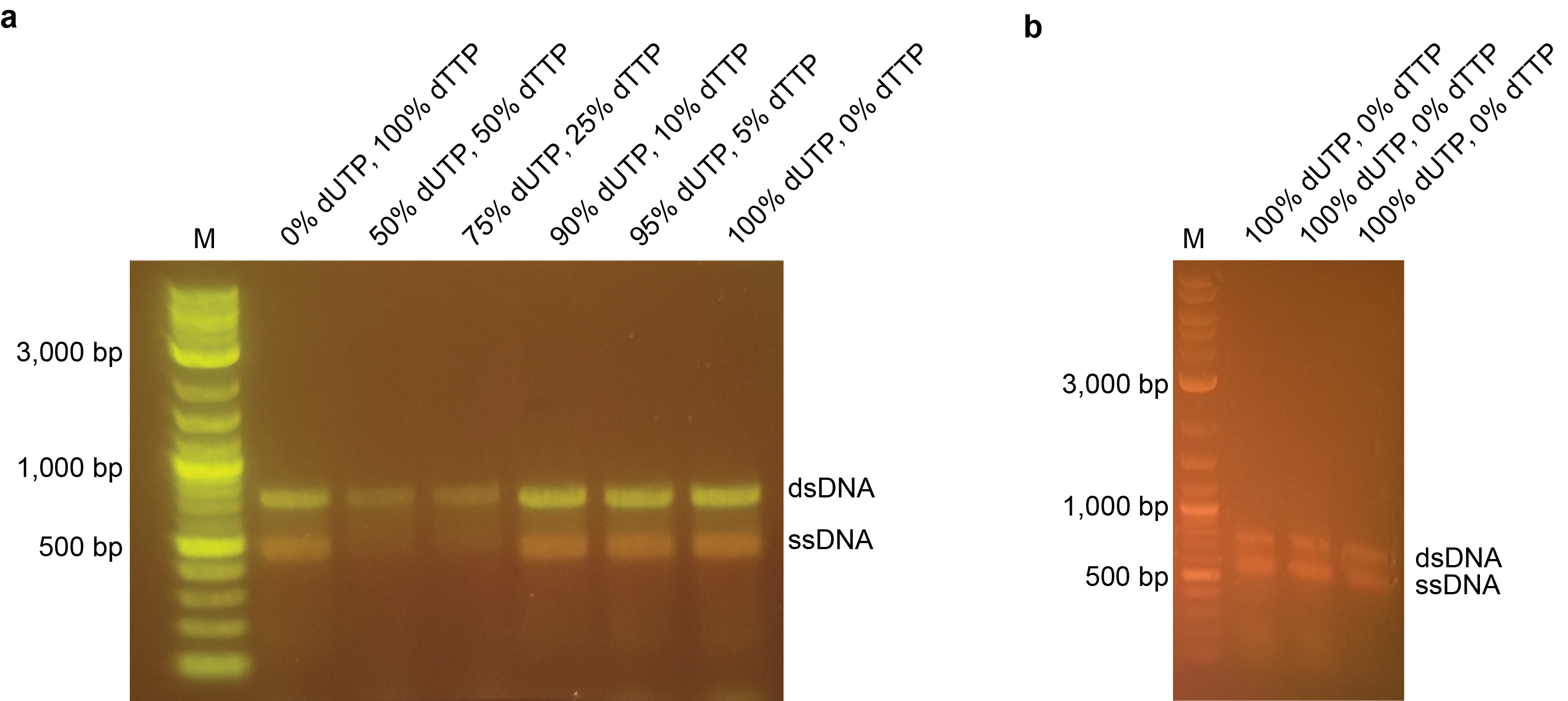


**Figure S17. Base replacement in asymmetric production of ssDNA. a.** Amplification of an mCherry ssDNA fragment (735 nt ssDNA) using gBlock as a template with percent replacement of dTTP with dUTP, as indicated in the associated lane. Agarose gel stained with SYBR Safe. **b.** Triplicate independent reactions of EGFP ssDNA with 100% replacement of dTTP with dUTP using asymmetric production. Agarose gel stained with ethidium bromide.


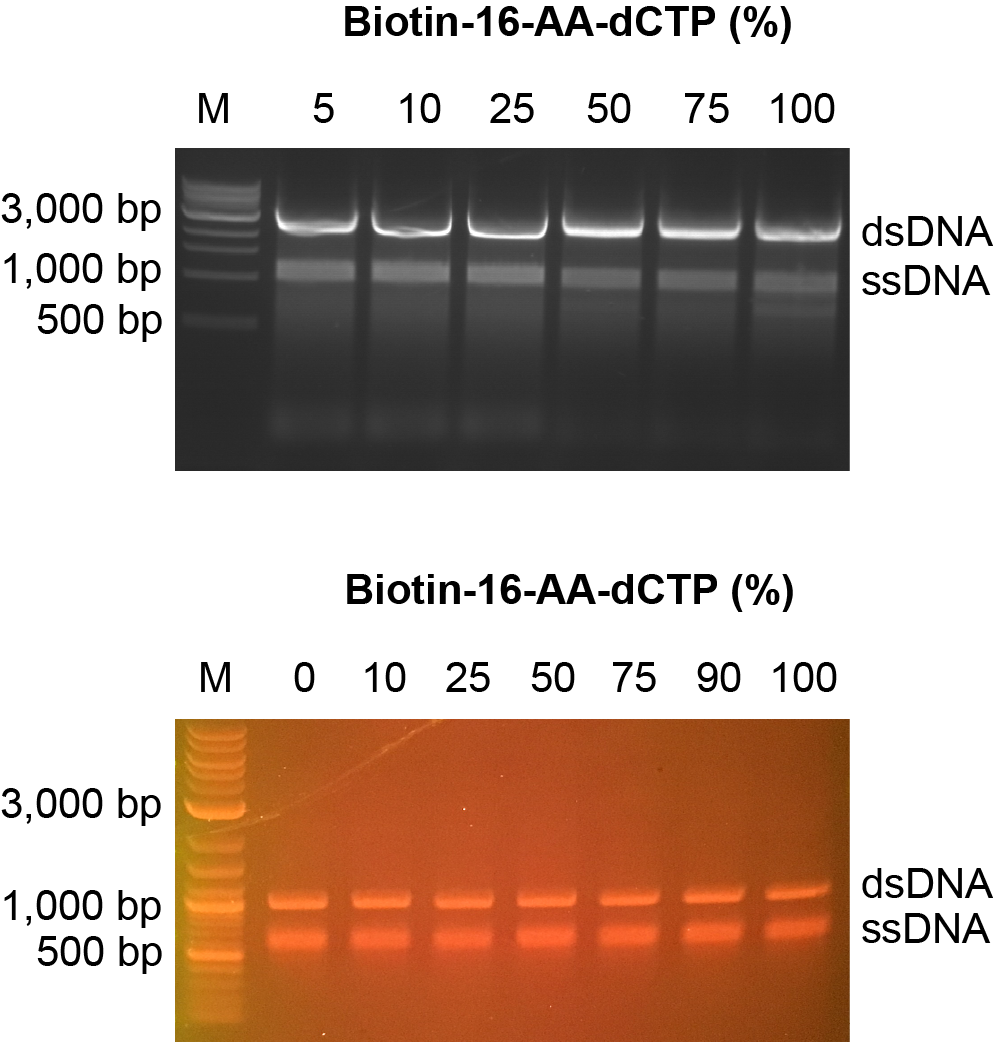


**Figure S18. Amplification of a 1,000 nt ssDNA fragment with increase concentration of Biotin modified dCTP.**


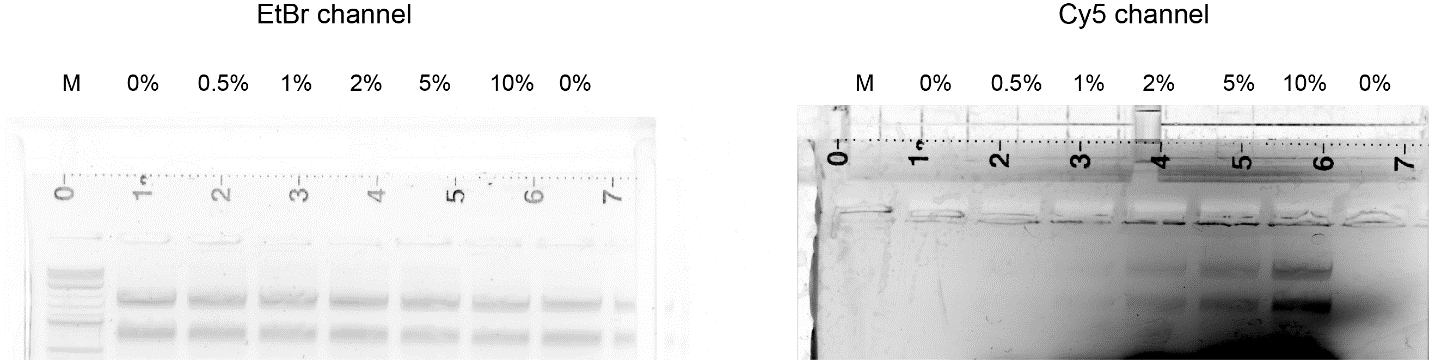


**Figure S19. Amplification of a 1,000 nt ssDNA fragment with increase concentration of Cy5 modified dCTP.** Image acquired with a fluorescent imager Typhoon 7000.

**Table S6. Amplification of a 1,000 nt ssDNA in pmole per PCR tubes (50 μL) for the different modified dNTPs tested.** Each sample represents average of 3 PCR tubes for each condition and the standard deviation.

| **Alpha Thiol dNTPs** | | | | |
| --- | --- | --- | --- | --- |
| **Percentage replacement** | **0%** | **50%** | **75%** | **80%** |
| **Average** | **2.33 ± 0.06** | **2.31 ± 0.15** | **1.84 ± 0.02** | **1.04 ± 0.46** |
| **Cy5 dCTPs (percentage replacement)** | | | | |
| **Percentage replacement** | **0%** | **5%** | **10%** |  |
| **Average** | **2.03 ± 0.31** | **2.19 ± 0.60** | **1.73 ± 0.16** |  |
| **Biotin dCTP (percentage replacement)** | | | | |
| **Percentage replacement** | **0%** | **25%** | **75%** | **100%** |
| **Average** | **2.33 ± 0.06** | **2.14 ± 0.10** | **2.07 ± 0.14** | **2.08 ± 0.07** |

**
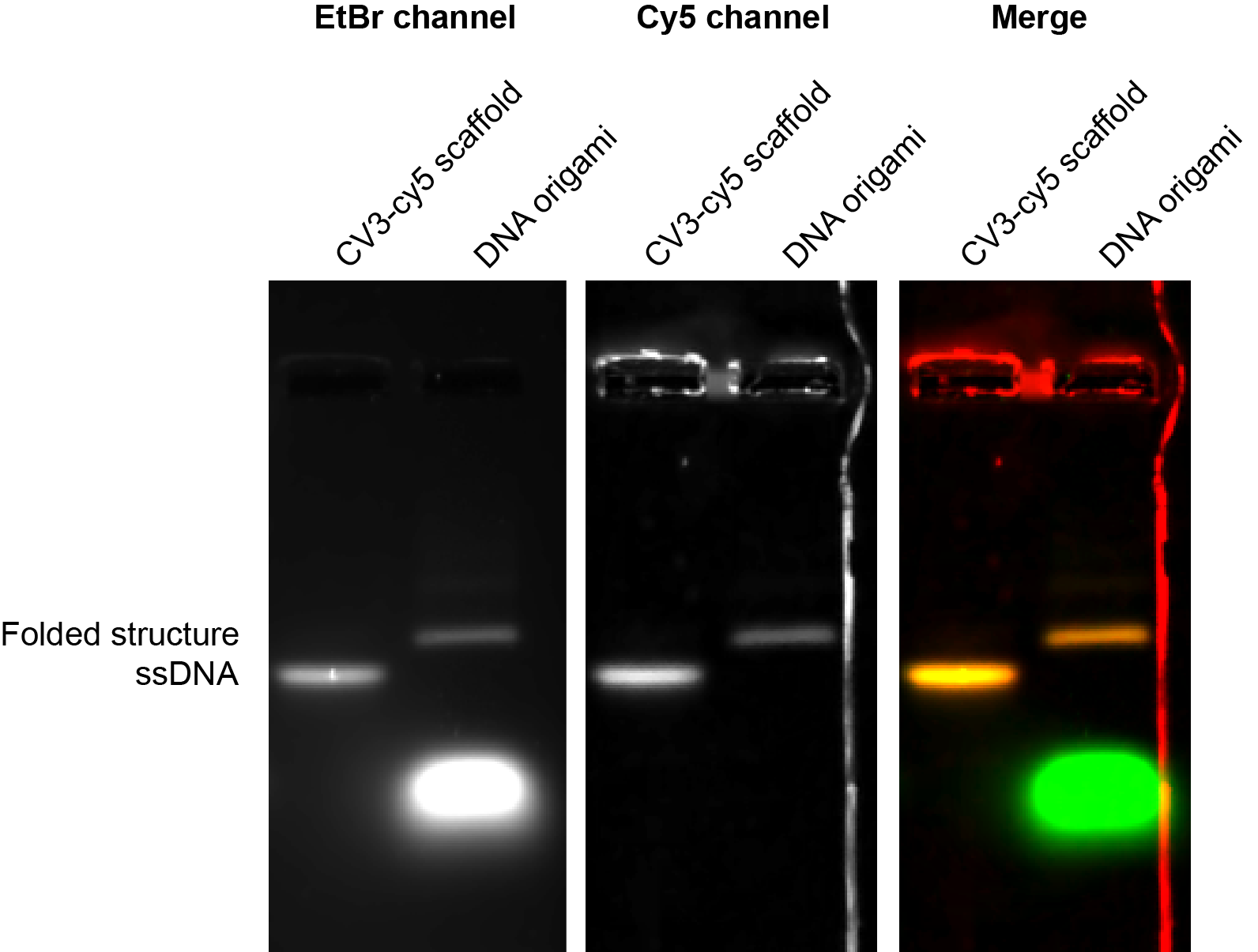
**

**Figure S20. Fluorescent 1,087 nt ssDNA (CV3) produced from aPCR spiked with 10% Cy5-dCTP used as a scaffold for folding a DNA origami tetrahedron.** Image acquired with a fluorescent imager Typhoon 7000. EtBr=ethidium bromide.

**
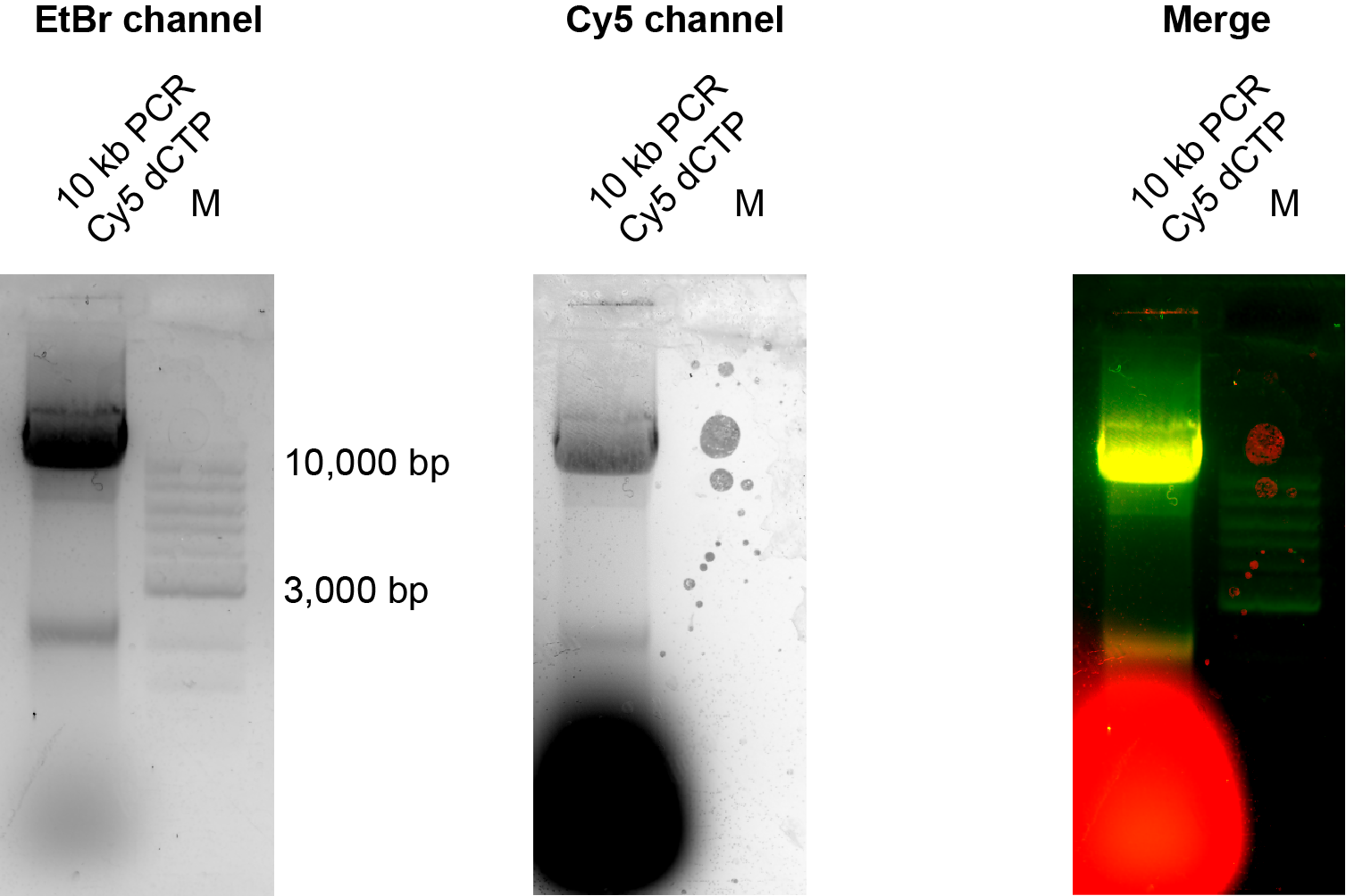
**

**Figure S21. Incorporation of Cy5-modified dNTPs in 10 kb ssDNA fragment.** 10% of modified Cy5-dCTPs are used to amplify a 10 kb fragment with aPCR. PCR product are run on a 0.7% agarose gel prestained with EtBr and imaged with a fluorescent imager Typhoon 7000.
